# Spatial capture-recapture design and modelling for the study of small mammals

**DOI:** 10.1371/journal.pone.0198766

**Published:** 2018-06-07

**Authors:** Juan Romairone, José Jiménez, Juan José Luque-Larena, François Mougeot

**Affiliations:** 1 Dpto. Ciencias Agroforestales, Escuela Técnica Superior de Ingenierías, Universidad de Valladolid, Avda. De Madrid, Palencia, Spain; 2 Instituto Universitario de Investigación en Gestión Forestal Sostenible (iuFOR), Avda. De Madrid, Palencia, Spain; 3 Instituto de Investigación en Recursos Cinegéticos (IREC, CSIC-UCLM-JCCM), Ronda de Toledo, Ciudad Real, Spain; University of Lleida, SPAIN

## Abstract

Spatial capture-recapture modelling (SCR) is a powerful analytical tool to estimate density and derive information on space use and behaviour of elusive animals. Yet, SCR has been seldom applied to the study of ecologically keystone small mammals. Here we highlight its potential and requirements with a case study on common voles (*Microtus arvalis*). First, we address mortality associated with live-trapping, which can be high in small mammals, and must be kept minimal. We designed and tested a nest box coupled with a classic Sherman trap and show that it allows a 5-fold reduction of mortality in traps. Second, we address the need to adjust the trapping grid to the individual home range to maximize spatial recaptures. In May-June 2016, we captured and tagged with transponders 227 voles in a 1.2-ha area during two monthly sessions. Using a Bayesian SCR with a multinomial approach, we estimated: (1) the baseline detection rate and investigated variation according to sex, time or behaviour (aversion/attraction after a previous capture); (2) the parameter sigma that describes how detection probability declines as a function of the distance to an individual´s activity centre, and investigated variation according to sex; and (3) density and population sex-ratio. We show that reducing the maximum distance between traps from 12 to 9.6m doubled spatial recaptures and improved model predictions. Baseline detection rate increased over time (after overcoming a likely aversion to entering new odourless traps) and was greater for females than males in June. The sigma parameter of males was twice that of females, indicating larger home ranges. Density estimates were of 142.92±38.50 and 168.25±15.79 voles/ha in May and June, respectively, with 2–3 times more females than males. We highlight the potential and broad applicability that SCR offers and provide specific recommendations for using it to study small mammals like voles.

## Introduction

Obtaining accurate estimates of population size or density is crucial for ecological studies and management of animals but is often a challenging task. The study of populations and communities of small mammals, especially those with a cryptic or elusive way of life, traditionally demand capture-recapture data obtained from live-trapping sessions. The most widely method used for estimating the abundance of small mammals is the classical capture-recapture (CR) modelling [1–3] and is considered as the “golden standard” compared with other indirect methods that estimate abundance based on activity signs [4]. CR modelling uses a proportion of the real number of animals in a given area, assuming that each animal has an equal probability of being captured. Animals captured during the first encounter are released back to the population, which is then resampled, and the proportion of tagged animals is used to estimate the population size. In this case, numerous capture occasions are required to achieve reliable population estimates [5].

Traditional CR models have, however, two main limitations: first the effective sampling area is imprecise, and second, there is heterogeneity in capture probabilities between traps, due to the locations of individuals´ activity centres [6–9]. Novel advanced methods, such as spatial capture-recapture (SCR) models, have been developed to solve these issues. SCR take inferences about population density from capture-recapture data and spatial information from the locations of captures and traps [3,10–12]. These models have a great potential and are increasingly used for studying large or medium size mammals such as elusive carnivores [8,13–17]. Despite their great potential, SCR models have so far been little used for the study of small mammals, such as voles [2,18,19]. Voles are key to the functioning of many ecosystems, are often problematic when over abundant (becoming agricultural pests) and constitute one of the most studied mammal groups [20–22]. Improving methodological precision is, therefore, paramount to provide reliable estimates of vole numbers and their dynamics.

All capture-recapture models (CR and SCR) have technical limitations that need to be addressed when setting up a study, as these will influence data quality and model performance. Technical limitations include animal mortality in traps, which can be very high in small mammals [23–25], and trap saturation when using single-catch devices [2,26,27]. In addition, SCR models require a specific spatial organization of traps in the study area that depends on individual movements [10,16,28]. We focus here on solving these three technical limitations in order to provide specific recommendations for the study of vole populations.

Sampling wild animals may inevitably result in the death of some captured individuals. Therefore, when experimental designs require individuals to be live-trapped, marked and recaptured, mortality can seriously compromise data collection [29] and bias the results of capture-mark-recapture studies [30]. The risk of mortality in traps can be high for small mammals, because of hypothermia, dehydration, stress or food shortage [24,31,32]. For instance, in a trapping study in Oregon (USA), small mammal mortality in traps ranged from 6.8% to 64.3% depending on species [33]. Another study in Wisconsin (USA) showed mortality rates in Sherman traps ranging from <5% in deer mice (*Peromyscus* spp.) to c.10% in *Arvicolinae* rodents and > 75% in shrews [23]. In western France, mortality of common voles (*Microtus arvalis*) in traps throughout a capture-mark-recapture study averaged 11% [34]. Such risks can be mitigated using devices added to traps, such as a wooden end-chamber filled with cotton [35], a nest box [36] or plastic box covering the trap [37] that can enhance the survival of captured individuals.

Trap saturation, which is the average proportion of traps that are occupied and therefore not available for a new capture at the end of a trapping occasion [26], is another important methodological limitation, particularly when population density is high, inducing for instance negative bias in density (*D*) estimates [38,39]. Trap saturation can be avoided by adding more traps [38] or increasing the frequency of trap check, so that the new captures improve the accuracy of the estimates [40].

Of particular importance in SCR are the home range size of the target species, and therefore the spatial organization of traps and the maximum distances between traps [13,16,28], which determine the resolution of the information obtained on individual movement [41, 42]. For instance, if the distance between traps is too large, no information on animal movements may be inferred, because a given animal will only be captured in one trap [43].

Each species may display additional and particular complexities for the estimation of model parameters, which can be included in the modelling process. Most vole species are difficult to detect. Low detection probabilities arise because of their burrowing life style—they spend a variable amount of their time underground [44] or because of specific foraging tactics [45,46]. Modelling allows to investigate the influence of different factors on capture probability [47, 48] such as differences between sexes or classes of individuals [49], differences in behaviour (degree of trap-happiness, i.e., willingness to re-enter a trap [50] or any influence from the trapping devices; neophobia; influence of residual odours from previous captures [51– 53]). These model considerations will provide useful information about the study species and population, and, at the same time, reduce uncertainty around density estimates.

In the present study, we report on specific 1) technical considerations such as: mortality in traps, trap saturation, and spatial organizations of traps, and 2) modelling considerations that take into account variation in detection probability (i.e. sex, time or behaviour) or movement. All these considerations should be taken into account for successfully applying different models for the study of small mammals in general. We provide and test the effectiveness of a technical improvement to reduce mortality during trapping (the addition of a nest box to single-capture traps) and consider the influence of using new versus previously used traps, the number and spatial organization of traps (trap spacing relative to individual movement) to minimize trap saturation and maximize spatial recaptures. SCR allows to obtain accurate estimates of population density (with an associated error) and other relevant structural and functional parameters in the studied population, such as sex-ratio, space use and movement. We illustrate this using an empirical study of a free-ranging population of common voles (*M*. *arvalis*) from an experimental field located in NW Spain.

## Material and methods

### Ethics statement

All the work with small mammals conducted by our research team, and leaded by Dr. Luque-Larena, has been approved (File number: 4801646) by the “Comité de Ética en Experimentación y Bienestar Animal (CEEBA)”, the relevant ethic committee of the University of Valladolid. We also held all the necessary permits to manipulate wild animals, as required by Spanish regulations in force (Real Decreto 53/2013).

### Study species and area

The common vole is a small sized herbivore (<100g) distributed from Northern Spain to the Middle East and Central Russia; it is one of the most abundant rodents in Europe and a facultative agricultural pest [54]. Some common vole populations are truly cyclical [55, 56], while other populations seem to fluctuate irregularly [54]. In northern and central Europe, multiannual population outbreaks are a common feature and occur regularly every 2–5 years [57]. Some common vole populations cause important crop damages and economic loss during outbreaks in farmland areas[54]. In addition, they act as reservoir and amplifier of zoonotic diseases that have important public health implications [58–61]. The study was carried out in a large intensive agricultural region of NW Spain (northern plateau, “Tierra de Campos”, Castilla-y-León region) where the farming landscape consisted of a mosaic of crops dominated by non-irrigated cereals [62].

### Nest box design and its influence on small mammal survival

In order to increase the survival rate of captured small mammals, we developed a nest box that was coupled to a classic Sherman trap (8 × 9 × 23 cm; LFTA Sherman). We created a device that has some operational similarities with the Longworth trap (Longworth Scientific Instrument Co., Oxford, England), which consists of a nesting chamber box coupled to a tunnel with a trigger and trap [63]. The Longworth trap is made of metal (usually aluminium), which provides poor insulation from cold or heat for the trapped animal. In a comparative study between live-trapping devices, Longworth traps had a reduced capture efficiency compared with Sherman traps in some environments [64]. Another study [65], comparing Longworth, Sherman and Uggland traps (Granhab, Gnosjö, Sweden), found a slight difference in capture efficiency between the three trapping devices, which was lower in Sherman traps and similar in Longworth vs. Uggland [65, 66]. Here, we used the Sherman trap, which is made of metal, foldable and has an efficient trapping mechanism, but we added to it a nest box made of thick plastic material to provide an additional shelter with better insulation for trapped animals. Each nest was made using a section of a P.V.C drainage plumbing tube (diameter of 125 mm, length of 150 mm and thickness of 3.75 mm). Both sides of the P.V.C tube were sealed with end caps, and in one cap a hole was cut following the shape and dimensions of the shortest side of a Sherman trap, allowing its coupling with the nest (see Figures A-D in S1 Supporting Information). The back door (i.e., the one located after the trap trigger) was removed to allow free movement of caught animals into the nest, while the front (trigger) door remained operational to prevent escape. During trapping sessions, nests were provided with dry bedding material (paper stripes) and baited with carrot, apple and rodent pellets (Global Diet 2018, Teklad).

We tested the effectiveness of the nest box at improving the survival of captured small mammals during trapping sessions of a seasonal monitoring program conducted in Tierra de Campos [62, 67]. This monitoring consisted of regular vole trappings every 4 months (in March, July and November) in fields of cereal, alfalfa or in fallows. During the capture sessions of November 2016, March 2017 and July 2017, we sampled 60 field margins (20 each month), a linear habitat feature that hosts a large proportion of the vole population [67]. We set up 10 traps in each field margin spaced every 2m and alternated traps with and without a nest (5 in each group; see Figure D in S1 Supporting Information). Traps were set in the morning and checked and retrieved after 24h. For each captured mammal, we recorded the species, if it was alive or dead and if the trap had a nest or not. We used these trapping data to compare the proportion of each small mammal species that was retrieved alive from traps with or without nest. Most captures (n = 430) were of common vole (66%), but we also captured wood mouse (*Apodemus sylvaticus*) (14%), the Algerian mouse (*Mus spretus*) (16%) and common weasel (*Mustela nivalis*) (4%). We also report on the effect of nests on the survival of these other species in traps.

### Experimental field and spatial capture-recapture study

The SCR study was conducted in May and June 2016 in an experimental field of 1.2 hectares managed by the Universidad de Valladolid. The experimental field is located in Soto de Cerrato (41° 94' N, 4° 42' W), Castilla-y-León region. The area is characterized by an intensive agricultural landscape with cereal, alfalfa, and fallows [62, 67]. The experimental field was planted with alfalfa (“Aragón” variety, non-irrigated) in spring 2015, and enclosed by a wire mesh fence of 2.5 m height to prevent access from people while allowing small mammals to move through it.

Common vole monitoring within the enclosure was carried out using a capture-mark-recapture method in a marked study grid. All trapping devices were classic Sherman traps fitted with a nest (as described above). Each trap was also covered by a large U-shape concrete block to protect it from heavy rain, direct sunlight and frost (Figure C in S1 Supporting Information).

**Fig 1:**
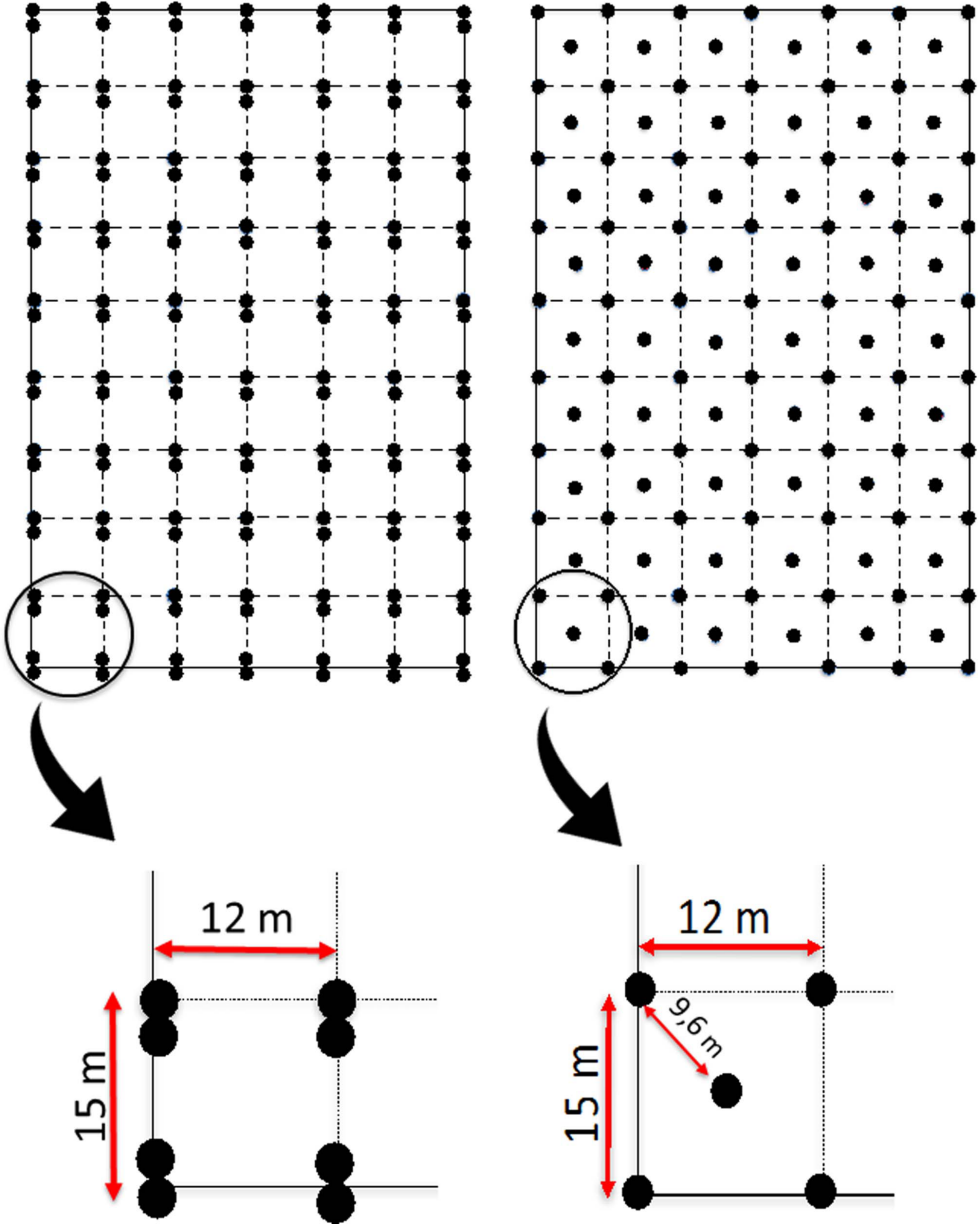
Spatial organization of the traps (grid design) used for the SCR study. In May (A) the trapping grid included 140 traps (black dots), set up in pairs at 70 points spaced by 12 or 15m. In June (B) the grid included 124 traps set up singly at 124 points, with distances between traps of 15, 12 and 9.6 m. The overall trapping array covered 9720 m^2^ (72 × 135m).

In May 2016, we set up a trapping grid consisting of 140 traps placed in pairs at 70 grid nodes regularly spaced (72 × 135 m, 9720 m^2^ area) within the experimental field, with a minimum distance between traps of 12m (Fig 1A). We set up two traps at each node in order to avoid trap saturation, which could result in poor model estimates [26]. We favoured single-capture traps because they were found to have a better trappability than multi-capture traps in arid environments [68] and Nearctic temperate regions [65]. In June 2016, the trapping grid was modified in order to reduce the minimum distance between traps and investigate effects on spatial recapture rate (see Results). The altered grid then consisted of 124 traps regularly spaced (Fig 1B) with one trap per node and a minimum distance between traps of 9.6 m. Preliminary analysis of the May data indicated that trap saturation was low and not an issue, but the information on vole movement indicated that the minimum distance between traps should be reduced (Fig 1B).

Capture sessions consisted of two sampling periods (May and June) separated by a 2-weeks interval without trapping (Fig E in S1 Supporting Information). Each period was organized in 4 consecutive days of trapping, 3 days without trapping, and another 4 consecutive days of trappings with a total of 8 capture occasions during each session. Traps were checked twice a day (9:00 a.m. and 4:00 p.m.). Each captured vole was individually marked with a transponder (Glass tag, BIOGLASS 8625, 2x12mm, Ref: ICAR 941), sexed, weighed with an electronic balance to the nearest 0.1g, tail length measured with a ruler to the nearest 1mm, and then released at where it was captured. We also kept the right ear tip of each tagged vole as a source of DNA for future studies. This also allowed us to confirm that none of recaptured voles with a cut ear had lost its transponder or could not be identified because of transponder failure.

### Statistical analyses

#### Nests and small mammal survival in traps

We tested for each species separately whether the presence or absence of a nest affected the probability of a captured small mammal to die in trap. For common voles, the only species for which we had a large sample size (n = 284), we modelled survival probability (1 = survived, 0 = died in trap) using a binomial mixed model that included the field margin identity as a random factor (to account for the non-independence of data from the same field margin), the factor Nest (presence = 1 *versus* absence = 0 of a nest), the Month and the interaction Nest × Month, to test if the influence of the nest on survival differed between months.

For all the other species, the limited sample size prevented us from implementing mixed-models that included the field margin identity as a random factor (that failed to converge). We thus report results of simpler binomial models (as for the common vole, but without random effect), in order to report the effect of nest on survival. These models without random effect included Month, and thereby broadly controlled for differences in seasonal weather conditions during trapping, but did not allow to control for variations in microclimatic conditions, at field margin level.

#### Model considerations and parameters/covariates evaluation

In order to consider sex differences in movement and home range [8], we tested models in which the movement parameter sigma σ (the rate at which detection probability declines as a function of distance) differed between sexes (M_σsex_). We also analysed models where the baseline detection rate (the probability of detecting an individual at its activity center), *λ*_0_, can vary depending on three covariates: 1) sex (M_sex_), 2) behaviour (M_b_), 3) time (M_t_). The time effect considered that *λ*_0_ varies among capture occasions, for instance due to a progressive adaptation of voles to the presence of traps over time. This is especially relevant given that small mammals may behave differently towards “new” than towards “previously used” traps. The behavioural effect considered the degree of boldness of voles towards traps after their first capture [69]. After first capture individuals may become "trap shy" (less likely to enter a trap) or "trap happy" (more likely to enter a trap), or indifferent [70]. Finally, one sex may be more detectable than other (because of differences in home range, activity rythms, or other traits), and this must be taken into account to improve parameter estimates and obtain more precise estimations of density and population sex-ratio [71]. Interactions between covariates (time, sex and behaviour) were not included in initial models because of sample size limitations (low number of number of recaptures; see Results).

#### Spatial capture-recapture modelling

We estimated density and abundance within the state space *S*, that is an explicit spatial region within which the sampling of individuals occurs. It includes the trapping grid (spatial array of traps), and a buffer to ensures sufficiently large to include all individuals potentially exposed to sampling. In order to develop the state space in SCR, we used the values of σ obtained from a preliminary analysis, and the buffer was created using a distance at least 2.5 times the σ parameter of the half–normal from SCR model, beyond which an individual external from the trapping area would not be detectable. In our case study, we used 30-m buffer amplification around the trapping grid.

Our capture devices are single-catch traps used during 8 capture occasions each month. Multi-catch or independent multinomial distribution are used when the capture probability of an individual in a trap is not independent of its capture probability in other traps, and when the capture of a given individual does not affect the capture of other individuals. This last condition may sometimes be violated when using single-catch traps, but a simulation study [39] showed that the multi-catch model is still adequate for single-catch traps when saturation is below 86% (which was the case in our study). To date, no likelihood function currently exists for single-catch traps [39] (but see [26]). We therefore fitted a multinomial model using a Bayesian approach, accounting for dead individuals [8,72] and carrying out model selection on the various models described above. We took into account mortality in traps by adding a binary matrix that indicated during which capture occasion an individual died, so that it was no longer considered afterwards. We favoured a Bayesian approach because in BUGS [73] the construction of even very complex models becomes relatively feasible, transparent and easy to understand [74]. The Deviance Information Criterion (DIC) is not appropriate for model selection with mixed models such as SCR models [75]. Here, we first evaluated if the sigma parameter differed between sexes. Then, using the selected model, we fitted all covariates that could potentially affect vole detection (time, behaviour and sex) using the logit function:

$$logit\left(p_{0}\left[i,k\right]\right)=\alpha_{0}+w\left[1\right]\cdot \alpha_{1}\cdot time\left[k\right]+w\left[2\right]\cdot \alpha_{2}\cdot C\left[i,k\right]+w\left[3\right]\cdot \alpha_{3}\cdot sex\left[i\right]$$

Where *i* are the individuals; *k* the occasions, and *C* a binary matrix that was used to account for differential behavioural responses of individuals to survey devices related to different capture histories, where *C*_*i*, *K*_ = 1 if individual *i* was captured at least once prior to session *k*, otherwise *C*_*i*, *K*_ = 0. We did not included interactions between covariates due to sample size limitations (number of recaptures and spatial recaptures; see Results). For model selection, we used the Kuo & Mallick [76] indicator variable (*w*) selection approach to select the best candidate model in relation to the use of both parameters in the models [8] and we evaluated the sensitivity of posterior model probabilities to different prior specifications using a normal distribution *N*(0, *σ*^2^), with *σ*^2^ = 10 and *σ*^2^ = 100. All these SCR models can also be fitted in a Maximum likelihood estimation framework using secr [77] or oSCR [78], with the advantage of using the Akaike Information Criterion [79] for model selection. We have also done this alternative model selection (see Table B in S1 Supporting Information).

Models were run in Nimble [80]. We ran 3 chains of the Markov Monte Carlo (MCMC) sampler with at least 150,000 iterations in each case (see Dataset in S2 and S3 Supporting Information). To check for chain convergence, we assessed MCMC convergence by visually inspecting trace plots for each monitored parameter, and we calculated the Gelman-Rubin statistic using the R package coda [81] where values below 1.1 indicated convergence. For all parameters in our models, R-hat was always <1.1. Details of the models (scripts and outputs) are given as Supplementary Information (Dataset in S2 and S3 Supporting Information).

#### Testing and simulations

In order to better understand the results and check for possible sources of error, we also used our data to 1) evaluate trap saturation levels at the scale of an average common vole home range and 2) to simulate the influence of the trapping grid configuration on key parameter estimates (abundance and σ).

To estimate saturation at the scale of common vole home range, we considered an average circular area of maximum detection of 2.5*σ radius [8] and used our trapping data in June to calculate for each trap and occasion the proportion of neighboring traps not available for capture within a < 2.5*σ distance around each trap (Table A in S1 Supporting Information).

For simulations, we randomly generated the locations of individual voles using the June model outputs (population density and σ) using a script modified from [8] and either the trap configuration used in May or the one used in June (see Fig 1 and S4 Supporting Information). We did four separate simulations (for each sex and trap configuration) using the Nimble software [80], with 100 simulated populations in each case, 5000 iterations and a 1000 burn-in. We determined the error in the estimated population size and σ, calculating the root mean square error (RMSE) of these study parameter for each sex and trap configuration.

## Results

### Effects of nests on small mammal survival in traps

**Fig 2:**
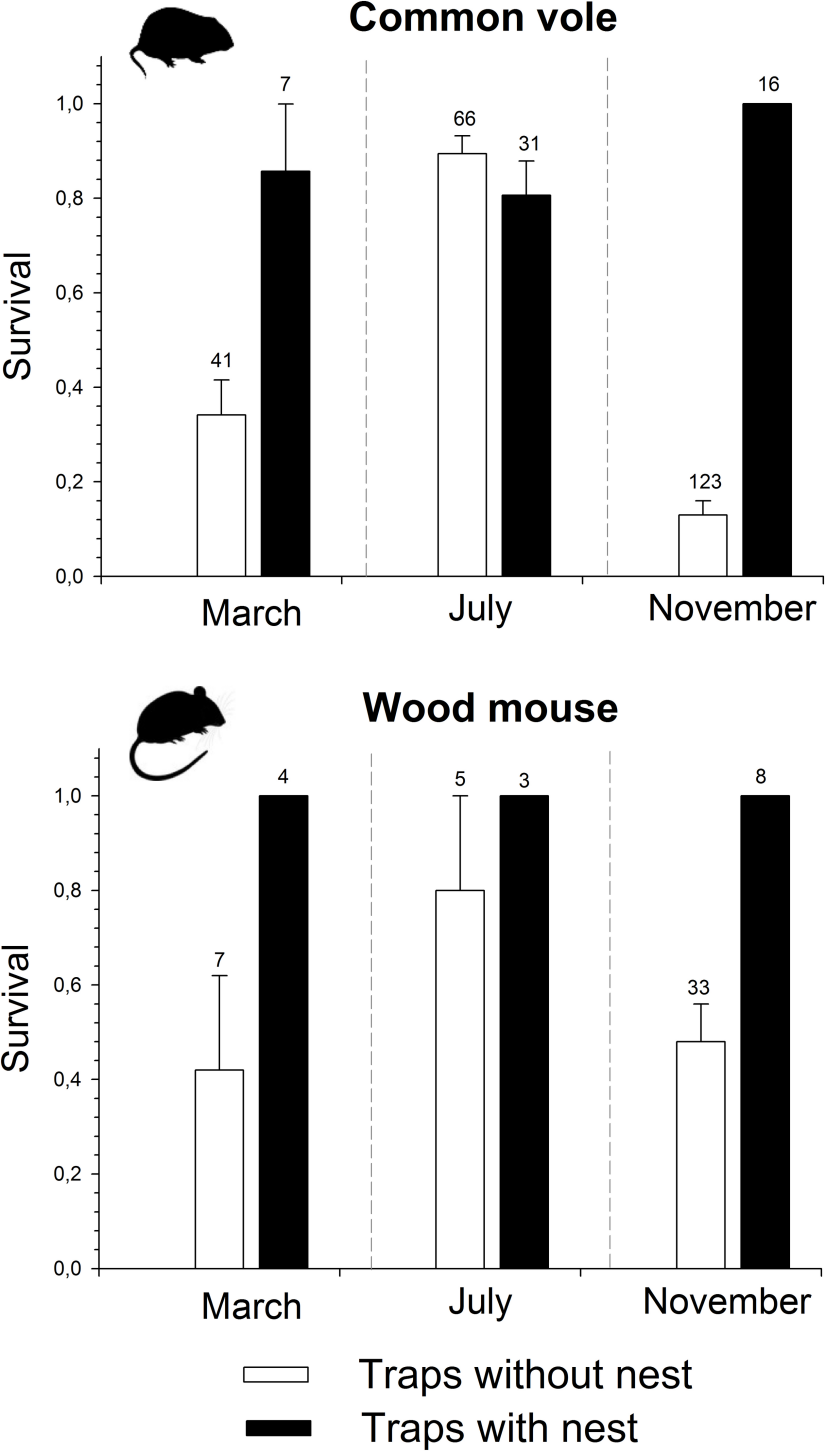
**Effect of the addition of a P.V.C nest box to a trap on the mean (± SE) survival probability of captured common voles (*Microtus arvalis*; A) and wood mouse (*Apodemus sylvaticus*; B) according to month.** Traps were set for 24h, alternating traps without nest (white bars) or with nest (black bars) in the same field margins. Number above error bars refer to the number of captured individuals.

We found a strong effect of adding a nest box on the survival of common voles during trapping, which differed between months (Mixed model; significant Month x Nest interaction: χ^2^ = 6.10; d.f. = 2, P = 0.047). In July, survival probability was highest and did not differ between traps with or without nest (χ^2^ = 1.32, d.f. = 1, P = 0.25; Fig 2A). In March, survival probability was significantly higher in traps with nests than in those without nests (χ^2^ = 6.82, d.f. = 1, P = 0.009; Fig 2A). In November, survival probability was overall lowest, and was also significantly higher in traps with nests than in traps without nests (χ^2^ = 54.90, d.f. = 1, P < 0.001; Fig 2A).

In the wood mouse, we found that survival probability was significantly higher in traps with than without a nest (χ^2^ = 15.80, d.f. = 1, P < 0.001; Fig 2B), with no significant differences between months (χ^2^ = 2.08, d.f. = 2, P = 0.354), and no significant interaction Month x Nest (Fig 2B). For the Algerian mouse we could only assess differences in survival in traps with or without nests in November (no individual was captured in traps with nests during March or July). Survival was lower in traps without nests (60.0 ± 7.4%, n = 45 captures) than in those with a nest (100%, n = 11; χ^2^ = 9.76, d.f. = 1, P = 0.0018). For the common weasel, we also found a similar pattern combining all months, with a lower survival in traps without nests (66.7 ± 14.2%; n = 12 captures) than in traps with nests (100%, n = 5).

### Vole captures in the experimental field

During the study, we made a total of 335 captures of common vole (106 in May and 229 in June) from 227 different individuals (81 in May, 1.31 captures/individual; 146 in June, 1.57 captures/individual). In May 66 individuals were detected once, 8 twice, 5 three times, 1 four times and 1 five times, with a total of 13 spatial recaptures. In June 103 inviduals were detected once; 21 twice; 13 three times; 4 four times, 3 five times and 2 seven times, with a total of 25 spatial recaptures. The number of captures and recaptures of male and female voles each month is reported in Table 1, describing the “naïve” vole population (as opposed to the modelled population; see below). The number of females increased considerably from May (n = 47) to June (n = 99), whereas the numbers of males slightly increased from May (n = 34) to June (n = 47). Common vole mortality in traps (which all had nests) averaged 2.98% (0.9% in May and 3.93% in June). The few individuals that died in traps were taken into account during the modelling (see Methods).

**Table 1:** Summary of the “naïve” population of common voles. Number of different males and females captured and marked during the sampling sessions of May and June 2016.

2016	Captured and marked (n)		
	Male	Female	Total
May	34	47	81
June	47	99	146

### Trap saturation and spatial recapture rates

With the original trapping grid design used in May (Fig 1A), we had 13 spatial recaptures (marked individuals captured in more than one trap during the session) and trap saturation (% of occupied traps in a given capture occasion) ranged between 0.71 and 8.57% depending on capture occasions. With the new trapping grid design used in June (Fig 1B), we had 25 spatial recaptures, while trap saturation ranged between 1.61 and 17.74%. Saturation values calculated within the maximum detection area (2.5*σ radius) around each trap for the all June capture occasions, are provided in Table A in S1 Supporting Information. These indicate that local saturation was never an issue as no trap had a consistently high saturation level over time (throughout the 8 capture occasions).

### Selection of covariates and model outputs

**Fig 3:**
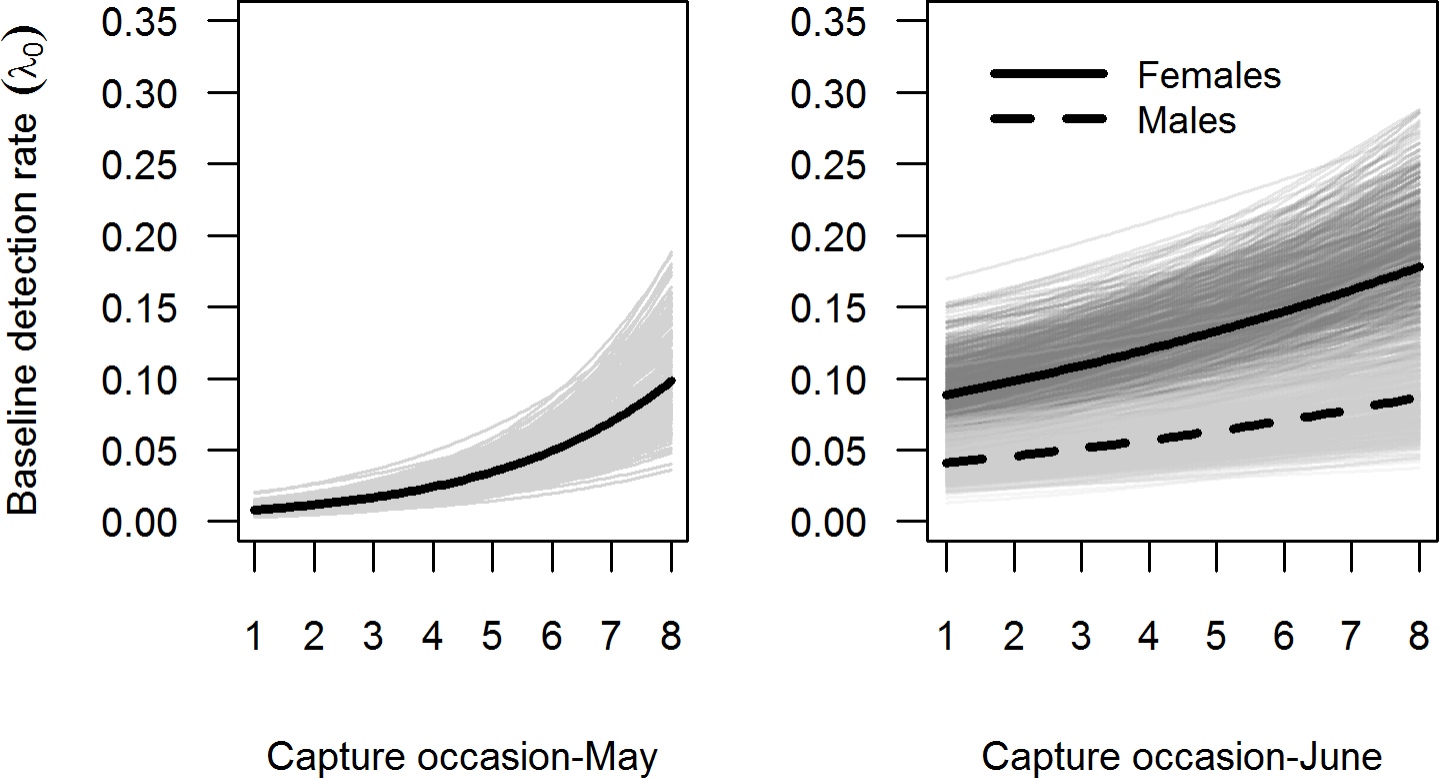
Changes over time in the baseline detection rate of common voles. In May (first capture session, left panel), detection rate increased over time with no difference between sexes. In June (second capture session, right panel), detection rate increase over time and was significantly higher for females (continuous black line) than for males (dashed black line). Thick black lines = posterior mean; thin grey lines = relationships based on a random posterior sample of 200 to visualize estimation uncertainty.

During the May capture session, we found no significant difference in *λ*_0_ between sexes and no significant effect of behaviour (Table 2). However, we found a significant effect of time (t) on *λ*_0_, which significantly increased between capture occasion 1 and 8 (Fig 3; Table 3) in both sexes. We also found that σ differed between sexes (Tables 2 and 3). σ_*females*_ averaged 5.94±1.19 (mean ± SD), while σ_*males*_ averaged 11.49±1.49 (Table 3), indicating greater movements and home ranges in males than in females. The density estimate, *D*, of females (96.22±36.41 individuals per ha) was almost twice that of males (46.70±8.66 individuals per ha), revealing a highly skewed sex-ratio (Table 3).

**Table 2:** Model selection for May and June. We first evaluated if the sigma parameter differed between sexes (see Fig F in S1 Supporting Information) and then used the Kuo & Mallick (1998) approach under two different priors to select covariates for *λ*_0_. The results are post-process model weights in a comparison of all possible models. Covariates: “sex” = male vs female; “b” = local behaviour or trap response by an individual; “t” = time covariate that varies with sampling occasion. Selected models are highlighted in bold.

	Parameters			Model weight comparison Kuo & Mallick (1998)	
	Priors	
Month	λ_0_			σ = 10	σ = 100
May	~1	~1	~1	0.000	0.000
	~1	~1	~b	0.000	0.000
	***~t***	***~1***	***~1***	***0*.*434***	***0*.*564***
	~t	~1	~b	0.374	0.359
	~1	~sex	~1	0.000	0.000
	~t	~sex	~1	0.112	0.044
	~t	~sex	~b	0.091	0.033
June	~1	~1	~1	0.000	0.000
	~1	~1	~b	NC	NC
	~t	~1	~1	0.160	0.359
	~t	~1	~b	NC	NC
	~1	~sex	~1	0.000	0.000
	***~t***	***~sex***	***~1***	***0*.*840***	***0*.*641***
	~t	~sex	~b	NC	NC

**Table 3:** Summary of posterior parameter estimates (mean ± SD) from the selected SCR models in May and June. Estimates were based on 3 chains of 150,000 iterations, thin rate = 1, and burn-in of 5,000 iterations, yielding 135,000 total samples from the joint posterior. BCI = Bayesian Credible Interval. Density = common voles/ha; D. females = females/ha; D. males = males/ha; σ = Rate at which detection probability declines as a function of distance, by sex; pi = male proportion; psi = probability of unobserved individual is a member of a population. alpha0 = coefficients for baseline probability of detection (*λ*_0_), given by sex in June. alpha2 = coefficients for the time effect on *λ*_0_.

				BCI		
Month	Parameter	Mean	SD	2.50%	50%	97.50%
**May**	Density	142.92	38.50	88.58	135.98	235.04
	D. females	96.22	36.40	46.62	88.96	184.14
	D. males	46.69	8.65	33.02	45.45	66.43
	alpha0	-3.53	0.31	-4.15	-3.52	-2.93
	alpha2	0.90	0.13	0.64	0.90	1.18
	pi	0.34	0.08	0.18	0.33	0.51
	psi	0.36	0.10	0.22	0.35	0.60
	σ females	5.94	1.19	4.07	5.78	8.66
	σ males	11.49	1.49	9.03	11.34	14.86
**June**	Density	168.25	15.79	140.63	167.06	202.02
	D. females	126.33	14.68	101.01	125.09	158.11
	D. males	41.91	5.74	32.24	41.56	54.39
	alpha0 (females)	-1.92	0.22	-2.37	-1.92	-1.48
	alpha0 (males)	-2.74	0.25	-3.26	-2.74	-2.24
	alpha2	0.28	0.08	0.10	0.27	0.45
	pi	0.25	0.03	0.18	0.24	0.33
	psi	0.43	0.04	0.35	0.43	0.52
	σ females	4.10	0.29	3.58	4.08	4.72
	σ males	8.12	0.79	6.76	8.04	9.86

**Fig 4:**
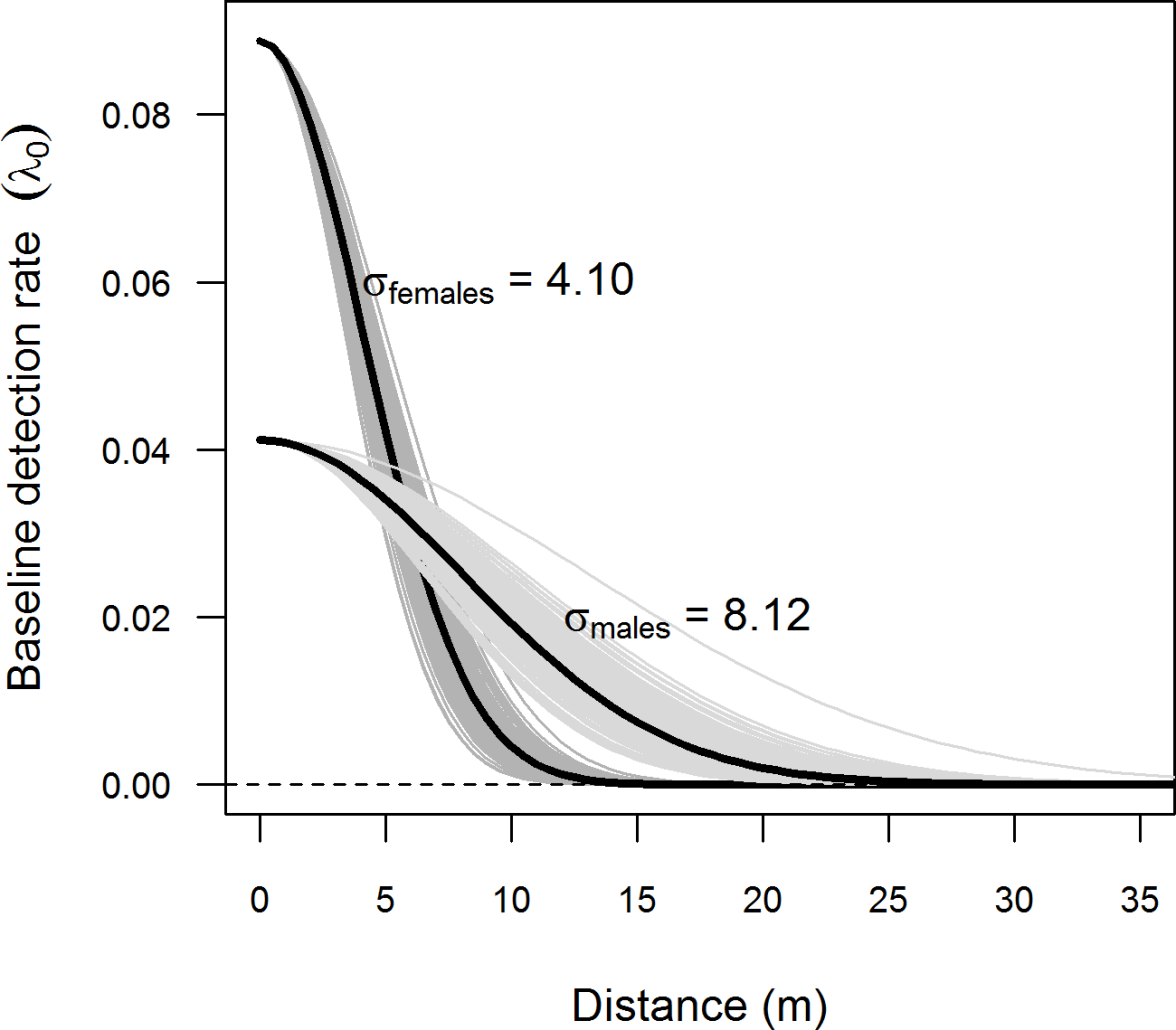
Relationship between the trap to activity center distance (m) and the detection rate of male and female common voles. The model outputs are for the primary capture session of June. The baseline probability detection (*λ*_0_) was significantly greater for females than for males, while the rate at which detection probability declined as a function of distance (sigma parameter) was c. 2 times greater for males than for females, indicating greater movements and home ranges. Thick black lines = posterior mean; thin grey lines = relationships based on a random posterior sample of 200 to visualize estimation uncertainty).

**Fig 5:**
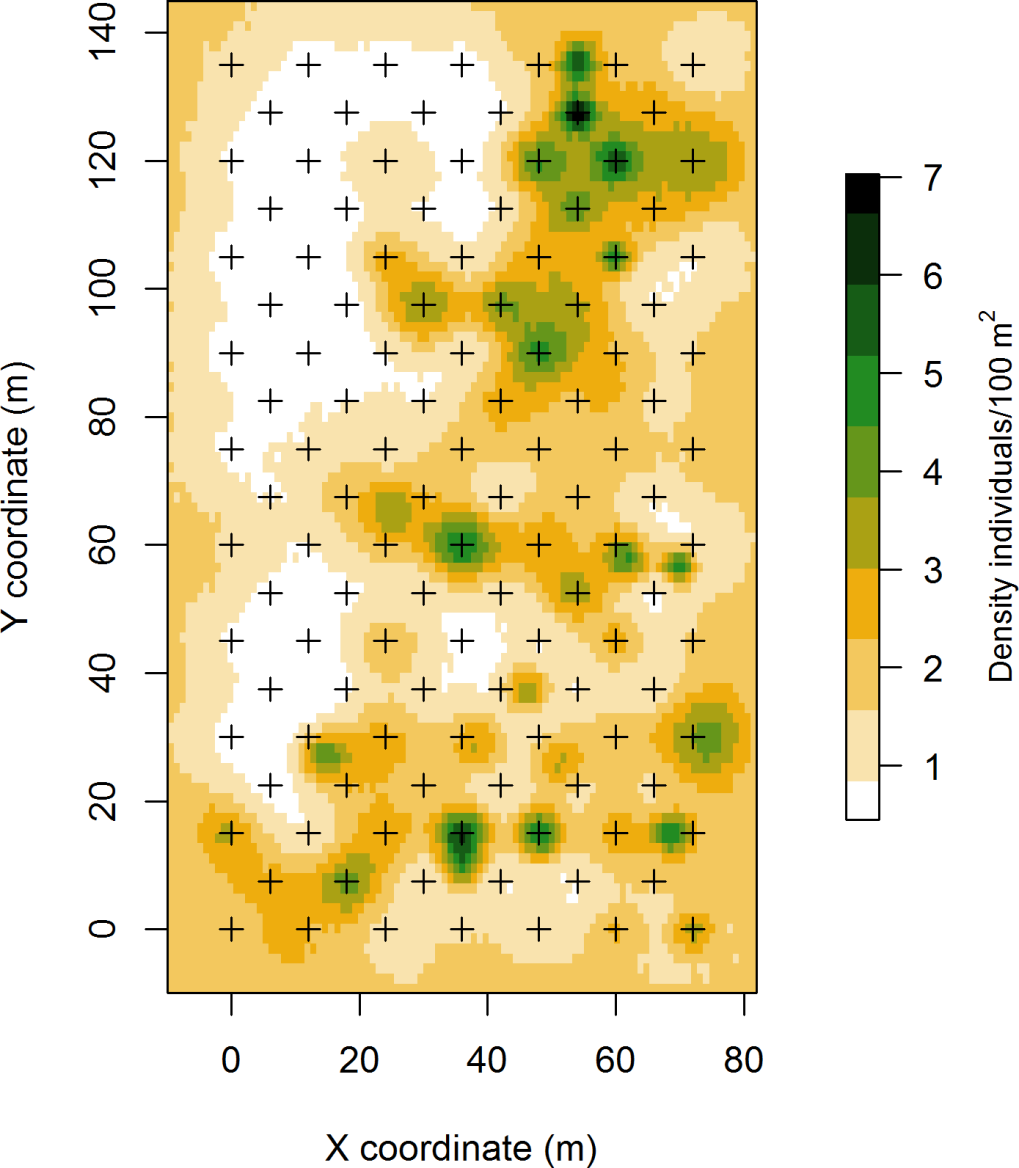
Predicted distribution and mean density of activity centres of common voles. The model outputs are for June. The state-space region covers 25740 m^2^, each pixel is of 1.45 m^2^ and the scale is in individuals/100 m^2^. Black crosses show the trap array, which covered 9720 m^2^ (72 × 135m).

During the June capture session, we could not test for a possible effect of behaviour in *λ*_0_ (models including behaviour could not converge; Table 2). We again found that *λ*_0_ increased over time (on the first capture occasion of June, *λ*_0_ was similar to the one of the last capture occasion of May, and continued to increase over time; Fig 3). Unlike in May, *λ*_0_ differed between sexes, being greater for females than for males (Fig 3; Tables 2 and 3). As for June, we found that σ differed between sexes, being almost double for males (σ _*males*_ = 8.12±0.79) than for females (σ _*females*_ = 4.10±0.29; Fig 4). Assuming a circular range around the activity centre [8] of an individual vole, the home range of males (1240 m^2^) was four times larger than that of females (316 m^2^). The density (*D*) estimate was 3 times larger for females (126.33±14.68 individuals per ha) than for males (41.91±5.74) (Table 3). A graphic representation of density across the state-space area, *S*, and trapping grid is shown in Fig 5.

Simulation analyses using population data from the June model showed that changing the trap configuration from the one used in May to the one used in June (with a reduction in minimum distance between traps from 12 to 9.6m but using the same theorical vole population) improved the precision of the parameters N^ (population size) and σ (movement; see Figure G in S4 Supporting Information). The reduction in root-mean-square errors (RMSEs) was particualry marked for the parameters estimated for females. This was because females had a lower σ than males (Table C and Figure H in S4 Supporting Information) and for these, the minimum distance between traps was initially too large, resulting in fewer captures and spatial recaptures.

## Discussion

### Trapping mortality

We first designed and tested a way to reduce mortality during trapping, by adding a nest to a single-catch trapping device (standard Sherman trap). We favoured the use of modified Sherman traps, rather than Longworth traps, in this study for two main reasons: 1) their practicality and effective trapping mechanism and 2) their reduced small mammal mortality due to the improvement of adding a non-metal nest box (mortality that was overall lower than that observed in other studies using standard Longworth traps [64]).

Mortality of small mammals in such traps can be very high, compromising our ability to follow individually marked individuals [23]. We have shown that using a P.V.C nest with food and bedding significantly reduced trapping mortality in common voles. Overall mortality was reduced 5-fold, but this effect was more pronounced in November and in March, when trapping mortality was also the greatest. By contrast, in July, trapping mortality was lower and similar in nests with or without traps (Fig 2). During our study, temperatures (mean [range: Min—Max]) were colder in November 2016 (5.8°C [-4.6°C to 21.7°C]) and March 2017 (8°C [-4.05°C to 24.9°C]) than in July 2017 (20.8°C [4.02°C to 35.4°C]), with some frost occurring during the former two months. This suggests that vole mortality was higher in colder weather conditions [82], in particular during frost, and that the addition of a P.V.C nest box minimized the risk of mortality by hypothermia. The characteristics (i.e. thick plastic walls) of our P.V.C nest boxes allows the captured individual to wait in an isolated warm and dry chamber until trap checking. Mortality could also increase during very hot conditions or under heavy rain because of trap flooding [33, 64]; we did not record any mortality due to heat or rain during our study, but their effect on survival with nests should be tested in future work. In another study in wet climate, mortality was reduced by protecting traps with a rain shield [33], although problems still arose when excessive rain flooded the bottom of the traps. Our study shows that the addition of a P.V.C nest box also improved the survival in traps of other species, such as the wood mouse, the Algerian mouse or the common weasel. These observations suggest that, under broadly similar weather conditions (same month), a coupled nest box should also perform well with these other small mammal species. However, a larger sample size would be desirable to test more rigorously the effect of the nest box on the survival of the latter two species, taking into account possible variations in microclimatic conditions at the field margin level.

### Trap saturation and spatial recapture rates

The number of traps and the frequency of visits to retrieve trapped individuals are key aspects to avoid trap saturation, i.e. a lack of active trapping devices available for new captures. In the present study, we initially set up trapping devices in pairs to avoid potential saturation. However, in May, with a density of about 140 voles per ha (Table 3), we always had less than 10% of occupied traps during a given capture occasion, so we were well below the previously recommended threshold of trap saturation of 60% [26] or 86% [39]. This allowed us to switch the design from pairs of traps per capture point to single traps [2], and to modify the grid (Fig 1) to try to increase spatial recapture rate, improve model performance and reduce uncertainty around parameter estimates.

In SCR studies, the spatial organization of traps and overall grid design should aim at increasing spatial recaptures (i.e. individuals captured in more than one trap in different occasions), to better inform about animal movement, and improve the accuracy of the model outputs. To set the distance between traps, a distance smaller than 2σ is recommend [16, 28]. We didn’t have any *a priori* knowledge of vole movement (σ) in our study area. After the session of May, spatial recapture rate was low (c. 5%) and the distance between traps appeared too large for an accurate estimation of σ (Fig 4). By adjusting the grid design (i.e., placing one trap in the middle of the initial square formed by four equidistant traps (Fig 1B), we reduced the minimum distance between traps from 12 to 9.6 m. With the new configuration of traps deployed in June, we obtained more captures (229 vs 106 in May), and more spatial recaptures (25 vs 13 in May). Overall, we also obtained measurements of σ and N^ with a reduced BCI in June compared with May (Table 3). The results of our simulations (S4 Supporting Information) indicated that the change in trap configuration contributed to improving the parameters estimates (reducing BCI). This was particularly evident for the parameter estimated for females (Table C in S4 Supporting Information), which had a lower sigma than males, and for which the minimum distance between traps was too large in May (Figure G in S4 Supporting Information) [28].

When trap spacing exceeds the average home range of a species there would be no or few spatial recaptures, as most individuals would be captured in a single trap, nearest their activity centre [43]. A primary aim when applying SCR models should be to achieve as many spatial recaptures as possible. Trap saturation in June increased to a maximum of about 18%, when traps were reduced from 140 to 124, but this was not an issue as saturation was kept to low levels. Saturation measured at the scale of an average area of maximum detection for a common vole was also not an issue. We never found a consistently high saturation level in any given trap throughout the 8 capture occasions (Table A in S1 Supporting Information). This is in agreement with a previous simulation study [39] that showed that the multi-catch model yielded nearly unbiased results for saturation levels below 86%, even when animals were clustered. If density increases and trap saturation becomes an issue, then the time interval between checking traps can be reduced and/or the number of traps increased.

### Insights from covariates retained in SCR model selection

During both May and June, the best selected model included a *λ*_0_ that increased over time (Table 3). An important component of capturing a particular individual is the chance that the trap will be encountered. For instance, the encounter rate can change due to the number of traps placed during the trapping period and their spatial placement [83]. In our study, we observed a clear lack of detection during the first capture occasions, and a progressive increase in *λ*_0_ throughout the following capture occasions. It is worth noting that in May we set up new, previously unused, Sherman traps and nests (Figure B in S1 Supporting Information). Previous works showed that odours left by previous occupants can affect subsequent occupancy, and traps with no previous occupant had lower capture rates [51]. The olfactory cues in and around a trap can strongly influence trap detection and entry by small mammals [84]. The complete absence of biological odours in new traps may therefore explain an initial neophobic response in voles, which typically show an active preference for substrates tainted with chemical signals from conspecifics [85–87]. We had no evidence for common voles to be trap-shy (less likely to enter a trap after an initial capture) or trap-happy (the opposite), at least in May when the behavioural effect could be included in the models. Hence, we suggest that the increased detection rate over time was mainly due to a progressive increase in olfactory cues left by the previously captured individuals (faeces, urine) that subsequently increased both trap detection and trapping rate.

In June, *λ*_0_ continued to increase over time, but also turned different between sexes (Fig 3): females ended up with a higher *λ*_0_ than males. This difference may be because female voles often have a socially dominant status [49] and/or are more territorial than males [88], but we do not have a clear explanation for such differences in detection rates between sexes. However, our result highlights that differential detectability between sexes shouldn’t be overlooked, as “naïve” estimates of male and female density could be consequently biased (i.e., in our case ignoring differences in *λ*_0_ or σ would result in an underestimated male density, because males were less detectable than females).

Interestingly, we found a marked difference in σ parameters between sexes during both months (Fig 4; Table 3). The estimated σ for males was twice that of females, indicating that males moved further away from their activity centre (burrow, colony) than females. SCR thus showed that females had smaller home ranges, were less mobile, but were still more likely to be detected. Our home range estimates (1240 m^2^ and 316 m^2^ for males and females, respectively) assume a circular home range with 2.5*σ radius around the activity center, which may be not very realistic in some cases (e.g. when the activity centre is located in a field margin instead of inside a field). Nevertheless, these estimates are consistent with those reported in other studies conducted on voles, like field voles *Microtus agrestis*, in which males (range 87–1037 m^2^) had larger home ranges than females (range 31–225 m^2^) [89]. Other studies on common vole home range reported a mean home range-size of 200 m^2^ [90], 145 m^2^ [91] or 125 m^2^ [92], without noticeable differences between sexes [90].

During the breeding season, females likely decreased their movements, while males increase theirs, probably in search of females to mate with. For instance, similar behaviour of active males searching females covering extensive areas at the beginning of the breeding season is also seen in other rodents such as meadow voles *M*. *pennsylvanicus* [93, 94], and the white-footed mice *P*. *leucopus* [95]. Understanding individuals´ movements in a given season or habitat is fundamental in ecological studies, and SCR method enables the integration of explicit movements with models that incorporate density and other population characteristics [18, 96].

### Model outputs

The SCR modelling allowed us to obtain a precise estimate of common vole density as well as information about how they were distributed within the study field (Fig 5). The species is colonial [22], and this is clearly evident from the density map provided as a model output. The model results also revealed a population sex ratio that was skewed towards females (with ratios of 2 and 3 females to 1 male in May and June, respectively). These estimates are accurate because they take into account differences between sexes in baseline detection rates and movements, two factors that can contribute to bias naïve estimates of common vole population sex ratio [71]. The sex ratio is a key parameter for population studies, but is difficult to reliably estimate it in voles. The SCR approach offers a credible and accurate estimate to investigate variation in this key population parameter. To ensure acurate estimates of sex-ratio, it is necessary to consider potential differences in movement between sexes, and to carefuly adjust the trap configuration (minimum distance between traps) to the movement parameter σ (sigma) of the sex that moves the less (see S4 Supporting Information). The application of SCR models allowed us to precisely estimate N^ (population size) and *D* (density), two crucial parameters for population dynamic or management studies [19,52]. In addition, SCR models allowed to describe important spatial characteristics of study species, revealing a different use of space by females and males (i.e. different home ranges) and provided a precise mapping of activity centres, allowing to clearly identify active burrows and colonies.

### Conclusions

SCR are relatively new models [8,10,12,39] that allow overcoming common challenges while providing a statistically coherent framework to estimate abundance and density. SCR models are increasingly used in ecological and conservation studies of mammals worldwide, but are still underused for studying small mammals like burrowing rodents [2,18,19]. After innovating, testing and adjusting the trapping methodology to our model species, we have highlighted the great potential and broader applicability that SCR modelling offers for studying voles. This should encourage further application of this methodology for the study of voles and other small mammals in order to get precise, reliable and accurate measurement of density and population structure, and to improve our understanding of their population ecology. To ensure accurate results, basic knowledge is however necessary to optimally design the study, and a pilot study may be particularly useful in the absence of detailed *a priori* knowledge on the species of interest. In order to successfully apply SCR to the study of small mammals such as voles, particular attention must be paid to: 1) reduce mortality in traps; 2) assess the possible effects of residual odours in traps (or the lack of these, when using new, unused traps) on capture rates; 3) monitor levels of saturation and adjust the number of traps or duration of capture occasion bouts accordingly; and 4) carefully adjust the trap configuration (minimum distance between traps) to the movement of the species, and more specifically to the class of individual that moves the less (like in our case females) in order to get more precise information on population composition (e.g. sex-ratio).

## Supporting information

S1 Supporting Information (DOCX)

S2 Supporting Information R script and model outputs for May. (HTML)

S3 Supporting Information R script and model outputs for June. (HTML)

S4 Supporting Information Simulation study. (DOCX)
