# Supplementary material for: Spatial capture-recapture design and modelling for the study of small mammals
Source: PLoS One. 2018 Jun 7;13(6):e0198766. doi: 10.1371/journal.pone.0198766 (PMC5991742; doi:10.1371/journal.pone.0198766)
Supplement: S1 Supporting Information — (DOCX) [file pone.0198766.s001.docx]

**S1 Supporting Information**

**Figure A.** **Photos illustrating the construction of the nest boxes added to the Sherman traps to reduce small mammal mortality in traps.** A) Design and cutting of P.V.C. tubes and end caps to fit the Sherman trap shape. B) Classic Sherman trap coupled with P.V.C. nest box.

A)


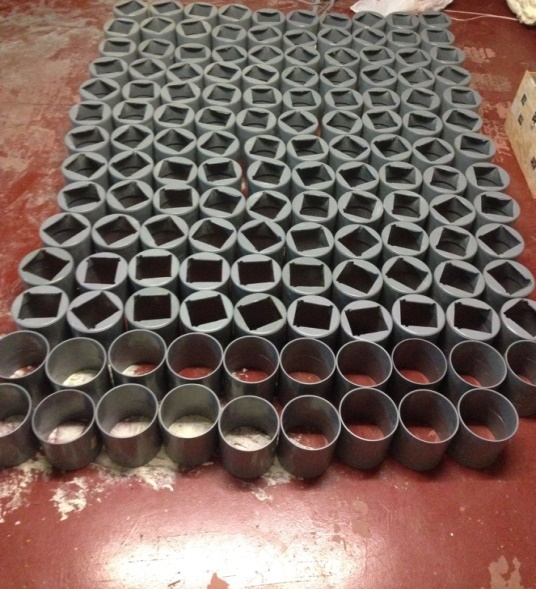

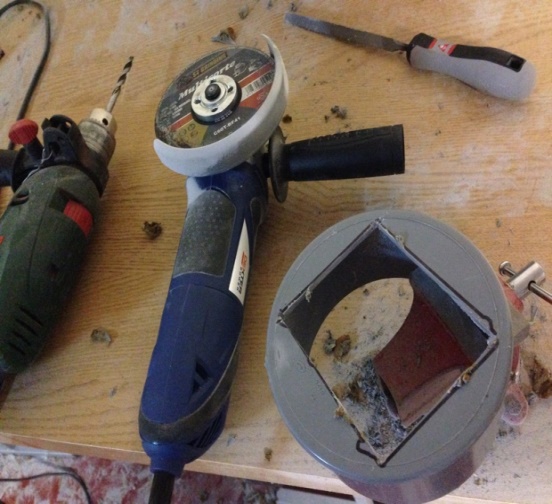


B)

| 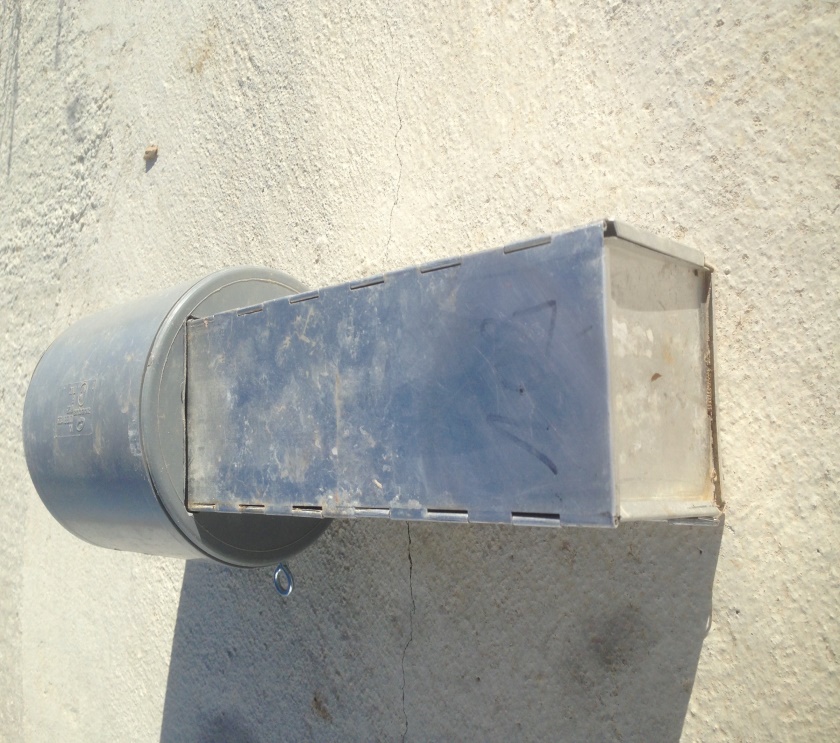 | 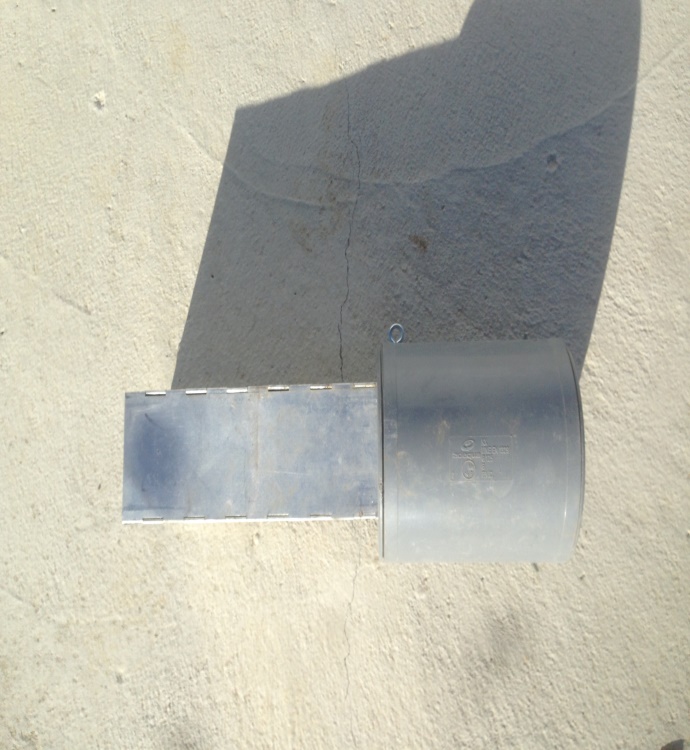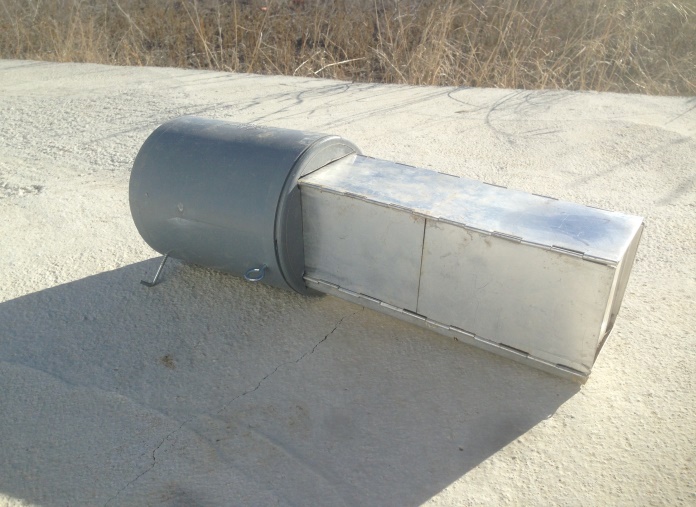 |
| --- | --- |

**Figure B. Photos of the Sherman traps coupled with their P.V.C nest boxes at the beginning of the study in May 2016.** Note that all traps were new and previously unused, which may explain their increasing capture rate over time.

| 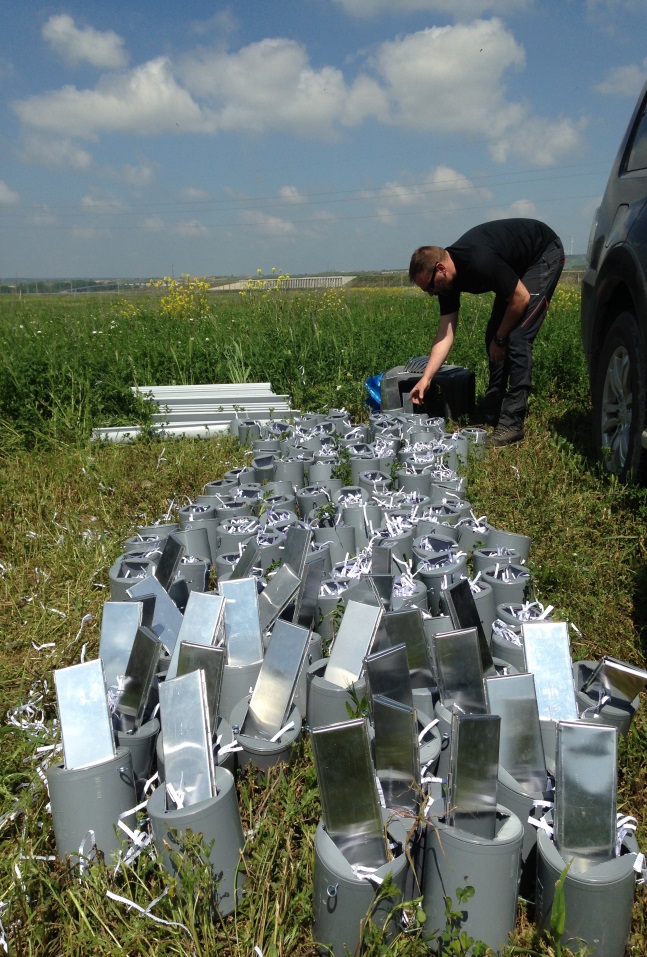 | 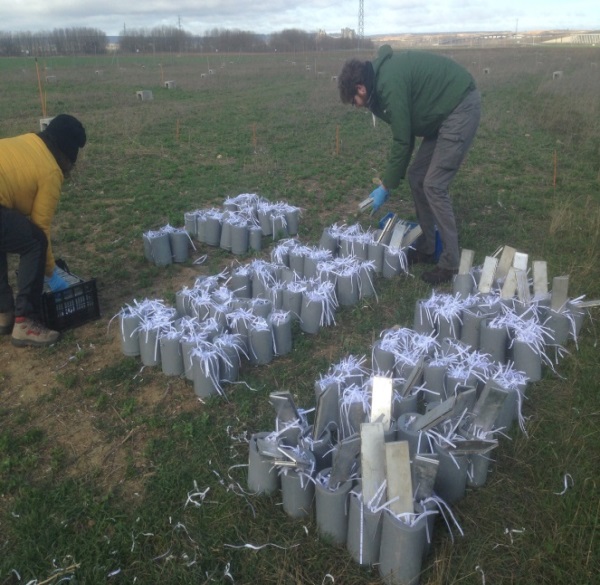 |
| --- | --- |

**Figure C**. **Photo showing the cement block placed upon each trap as an additional protection (from rain and direct sunlight).**

| 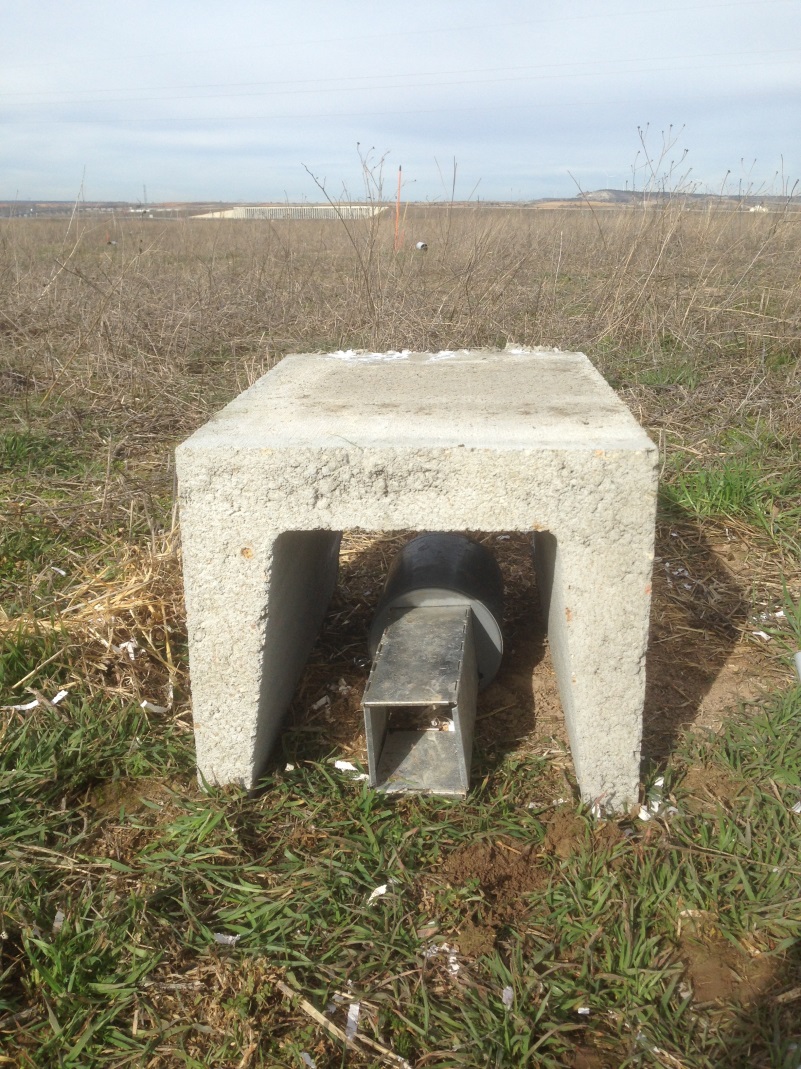 |
| --- |

**Figure D**. **Photos of the nest boxes designed and used in this study.** A) Captured common vole inside the nest (with its bedding material and food), B) Sampling design to test the effect of nests on trapping mortality, in which 10 traps were set in a field margin, alternating traps with and without a nest (5 of each; see methods).

A)


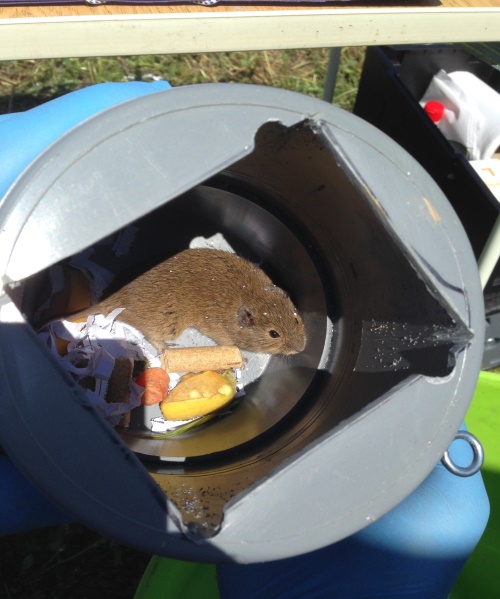


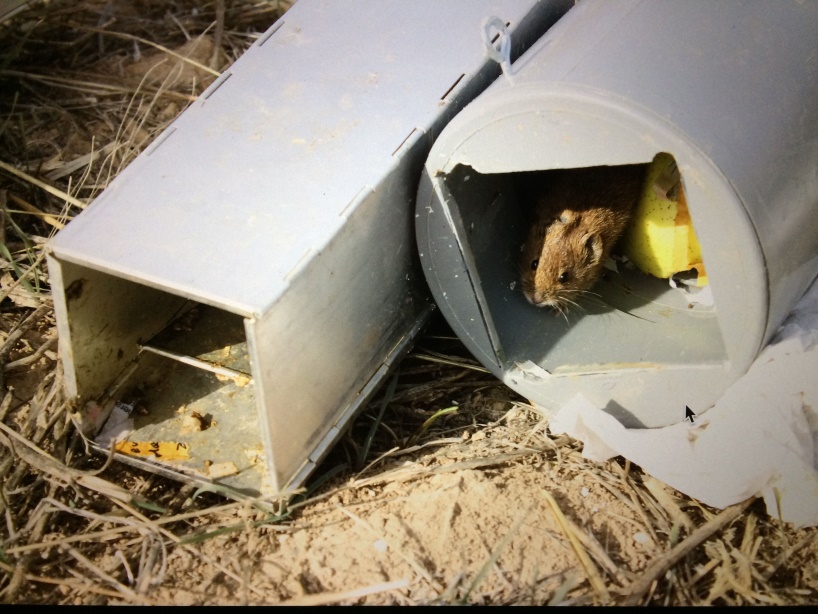


B)


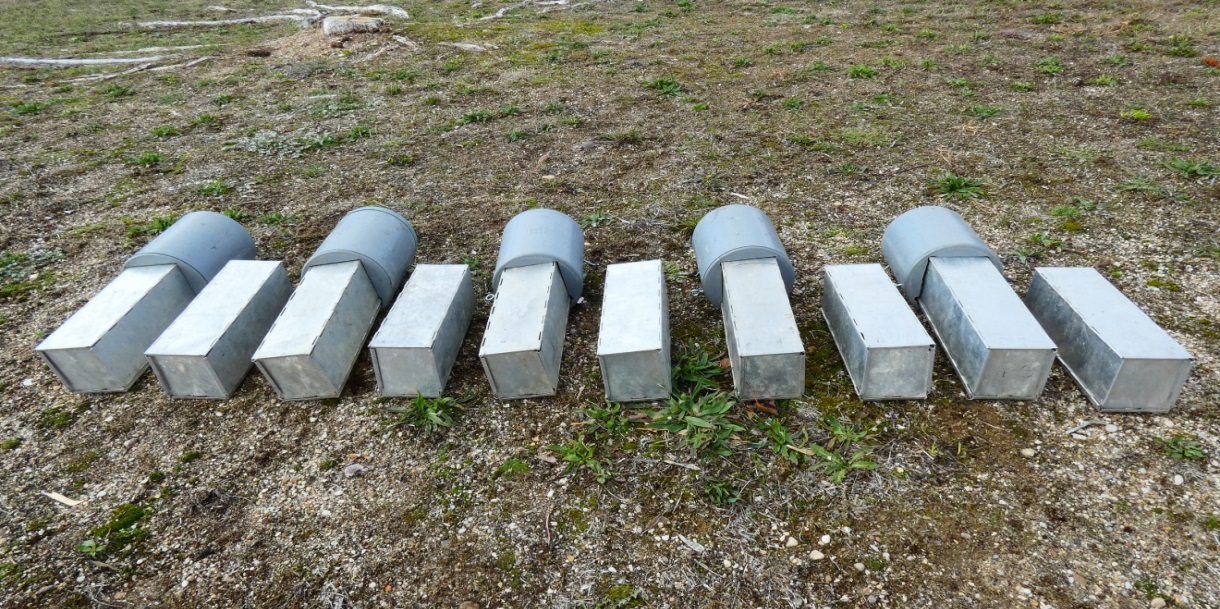

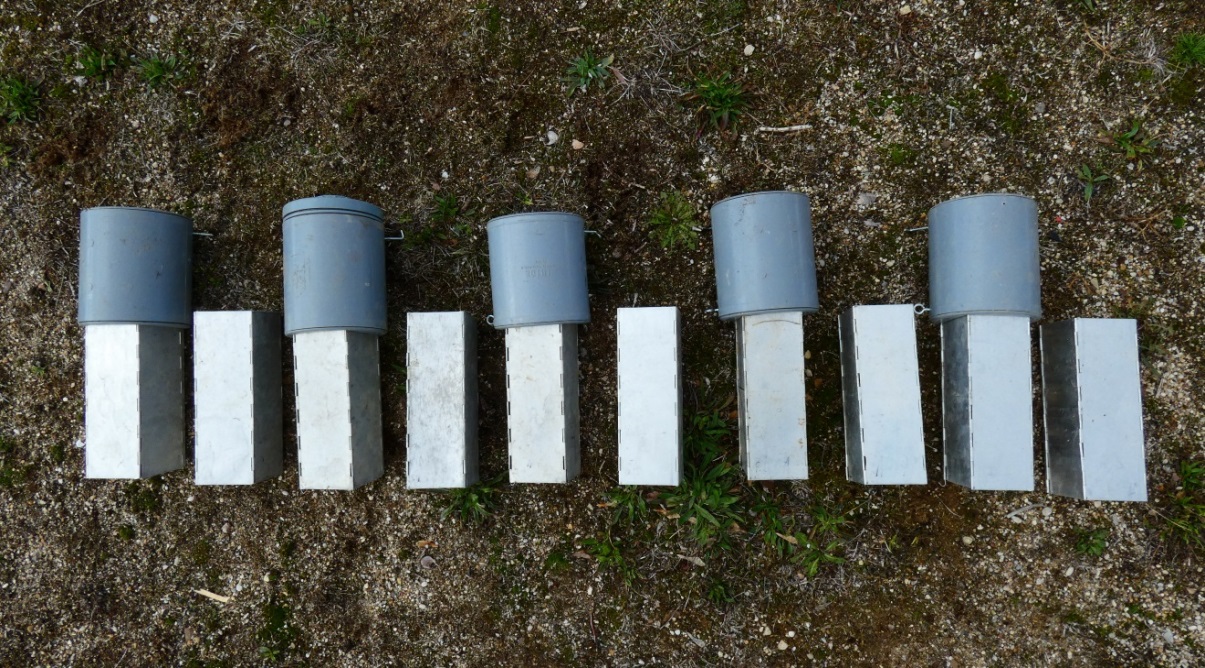


**Figure E. Chronogram showing the temporal organization of the trapping sessions.**

**Figure F.** **Model selection for sigma (σ; rate at which detection probability declines as a function of distance) in May (left) and June (right), considering sexual differences (male-female) in model parameters estimates.** There was a significant difference between sexes in σ in May and June.


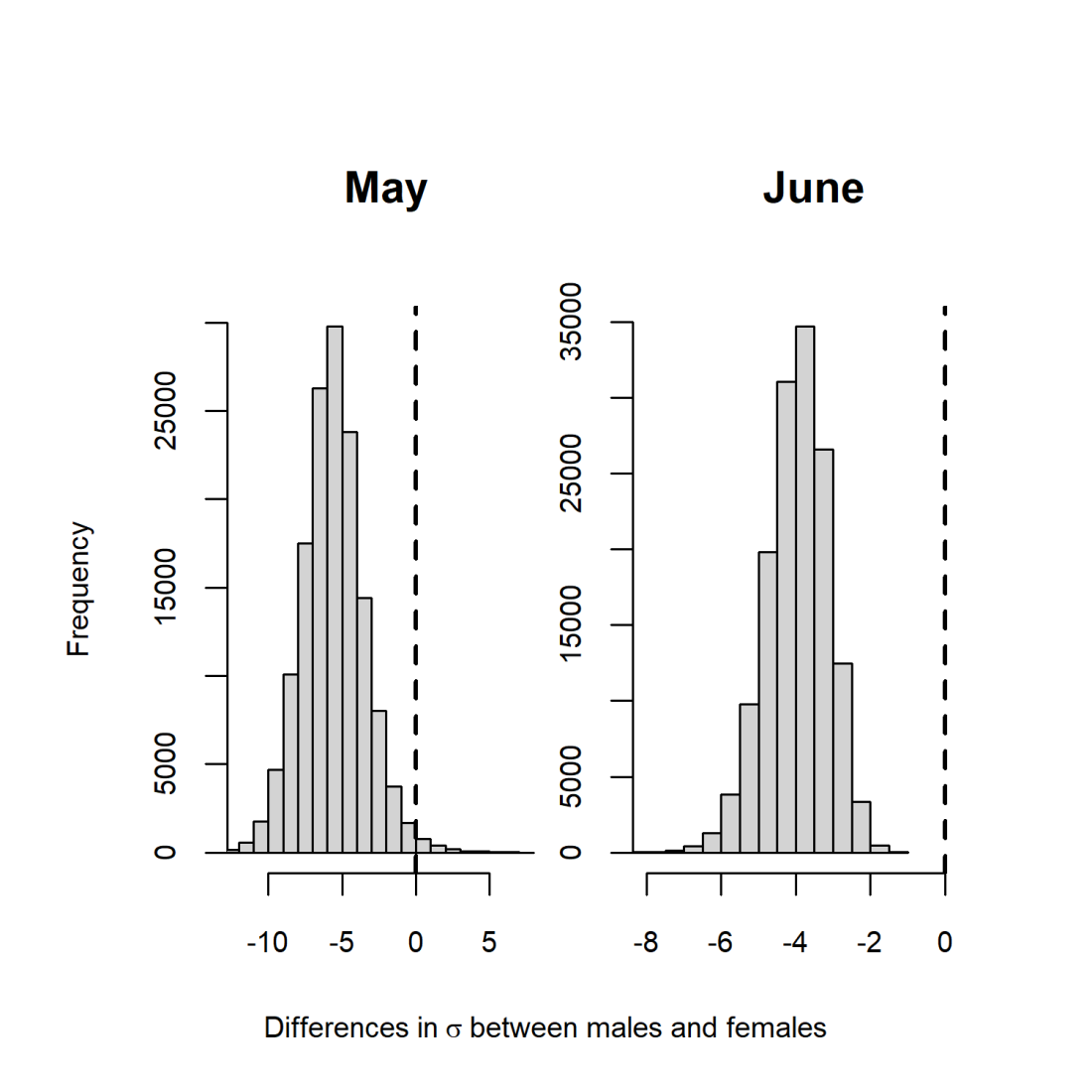


**Table A.** **Saturation calculated for each trap at an average vole home range level during June.** The Table shows for each trap and each occasion the proportion of neighboring traps within a 2.5*σ radius that were occupied and therefore not available for capture during a given occasion. No trap had a repeatedly high saturation over time.

| Trap ID | Occasion | | | | | | | |
| --- | --- | --- | --- | --- | --- | --- | --- | --- |
|  | 1 | 2 | 3 | 4 | 5 | 6 | 7 | 8 |
| 1 | 0.00 | 0.00 | 0.00 | 0.00 | 0.00 | 0.00 | 0.00 | 0.33 |
| 2 | 0.00 | 0.00 | 0.00 | 0.00 | 0.00 | 0.00 | 0.00 | 0.33 |
| 3 | 0.00 | 0.00 | 0.00 | 0.00 | 0.00 | 0.00 | 0.00 | 0.20 |
| 4 | 0.00 | 0.00 | 0.00 | 0.00 | 0.00 | 0.14 | 0.00 | 0.00 |
| 5 | 0.00 | 0.00 | 0.00 | 0.00 | 0.00 | 0.00 | 0.00 | 0.00 |
| 6 | 0.00 | 0.00 | 0.00 | 0.00 | 0.00 | 0.14 | 0.00 | 0.00 |
| 7 | 0.20 | 0.20 | 0.00 | 0.00 | 0.20 | 0.00 | 0.20 | 0.00 |
| 8 | 0.43 | 0.29 | 0.14 | 0.14 | 0.29 | 0.29 | 0.29 | 0.00 |
| 9 | 0.60 | 0.40 | 0.20 | 0.00 | 0.60 | 0.40 | 0.60 | 0.20 |
| 10 | 0.71 | 0.43 | 0.29 | 0.29 | 0.57 | 0.57 | 0.71 | 0.29 |
| 11 | 0.60 | 0.40 | 0.20 | 0.00 | 0.60 | 0.60 | 0.80 | 0.20 |
| 12 | 0.67 | 0.33 | 0.33 | 0.17 | 0.33 | 0.67 | 0.83 | 0.50 |
| 13 | 0.33 | 0.00 | 0.00 | 0.00 | 0.33 | 0.67 | 0.67 | 0.33 |
| 14 | 0.50 | 0.25 | 0.25 | 0.25 | 0.00 | 0.50 | 1.00 | 0.75 |
| 15 | 0.50 | 0.33 | 0.50 | 0.50 | 0.33 | 0.33 | 0.67 | 0.67 |
| 16 | 0.57 | 0.29 | 0.43 | 0.43 | 0.43 | 0.71 | 0.86 | 0.43 |
| 17 | 0.57 | 0.29 | 0.43 | 0.57 | 0.57 | 0.29 | 0.43 | 0.43 |
| 18 | 0.43 | 0.29 | 0.43 | 0.43 | 0.43 | 0.43 | 0.43 | 0.14 |
| 19 | 0.29 | 0.00 | 0.14 | 0.29 | 0.43 | 0.29 | 0.29 | 0.00 |
| 20 | 0.14 | 0.00 | 0.00 | 0.14 | 0.14 | 0.29 | 0.14 | 0.00 |
| 21 | 0.14 | 0.14 | 0.00 | 0.00 | 0.14 | 0.14 | 0.14 | 0.00 |
| 22 | 0.00 | 0.00 | 0.00 | 0.00 | 0.00 | 0.14 | 0.14 | 0.00 |
| 23 | 0.14 | 0.14 | 0.00 | 0.00 | 0.00 | 0.14 | 0.14 | 0.00 |
| 24 | 0.00 | 0.00 | 0.00 | 0.00 | 0.00 | 0.14 | 0.00 | 0.14 |
| 25 | 0.00 | 0.00 | 0.00 | 0.00 | 0.00 | 0.00 | 0.00 | 0.17 |
| 26 | 0.00 | 0.00 | 0.00 | 0.00 | 0.00 | 0.00 | 0.00 | 0.25 |
| 27 | 0.00 | 0.00 | 0.00 | 0.00 | 0.00 | 0.00 | 0.00 | 0.00 |
| 28 | 0.00 | 0.00 | 0.00 | 0.00 | 0.00 | 0.00 | 0.00 | 0.00 |
| 29 | 0.14 | 0.14 | 0.00 | 0.00 | 0.00 | 0.00 | 0.00 | 0.00 |
| 30 | 0.14 | 0.29 | 0.14 | 0.14 | 0.14 | 0.14 | 0.00 | 0.00 |
| 31 | 0.14 | 0.29 | 0.14 | 0.14 | 0.14 | 0.14 | 0.14 | 0.00 |
| 32 | 0.29 | 0.43 | 0.29 | 0.14 | 0.29 | 0.29 | 0.14 | 0.14 |
| 33 | 0.43 | 0.43 | 0.29 | 0.14 | 0.43 | 0.29 | 0.29 | 0.14 |
| 34 | 0.57 | 0.43 | 0.29 | 0.14 | 0.43 | 0.43 | 0.43 | 0.29 |
| 35 | 0.57 | 0.29 | 0.29 | 0.29 | 0.57 | 0.43 | 0.43 | 0.43 |
| 36 | 0.71 | 0.43 | 0.29 | 0.14 | 0.43 | 0.29 | 0.57 | 0.43 |
| 37 | 0.43 | 0.14 | 0.29 | 0.29 | 0.43 | 0.29 | 0.43 | 0.43 |
| 38 | 0.50 | 0.33 | 0.17 | 0.17 | 0.17 | 0.33 | 0.50 | 0.33 |
| 39 | 0.25 | 0.25 | 0.25 | 0.25 | 0.25 | 0.00 | 0.25 | 0.50 |
| 40 | 0.25 | 0.25 | 0.00 | 0.00 | 0.00 | 0.25 | 0.50 | 0.00 |
| 41 | 0.33 | 0.17 | 0.00 | 0.00 | 0.17 | 0.50 | 0.33 | 0.33 |
| 42 | 0.43 | 0.29 | 0.00 | 0.00 | 0.00 | 0.29 | 0.57 | 0.29 |
| 43 | 0.29 | 0.14 | 0.00 | 0.14 | 0.29 | 0.14 | 0.43 | 0.29 |
| 44 | 0.43 | 0.29 | 0.14 | 0.14 | 0.14 | 0.29 | 0.71 | 0.57 |
| 45 | 0.14 | 0.14 | 0.00 | 0.14 | 0.14 | 0.00 | 0.29 | 0.43 |
| 46 | 0.29 | 0.43 | 0.29 | 0.29 | 0.29 | 0.29 | 0.43 | 0.29 |
| 47 | 0.00 | 0.00 | 0.00 | 0.29 | 0.00 | 0.00 | 0.29 | 0.43 |
| 48 | 0.00 | 0.14 | 0.14 | 0.14 | 0.14 | 0.14 | 0.29 | 0.00 |
| 49 | 0.00 | 0.00 | 0.00 | 0.14 | 0.00 | 0.00 | 0.29 | 0.14 |
| 50 | 0.00 | 0.00 | 0.00 | 0.00 | 0.00 | 0.00 | 0.00 | 0.00 |
| 51 | 0.00 | 0.00 | 0.00 | 0.00 | 0.00 | 0.00 | 0.17 | 0.00 |
| 52 | 0.00 | 0.00 | 0.00 | 0.00 | 0.00 | 0.00 | 0.00 | 0.00 |
| 53 | 0.00 | 0.00 | 0.00 | 0.00 | 0.00 | 0.00 | 0.25 | 0.00 |
| 54 | 0.00 | 0.00 | 0.17 | 0.00 | 0.17 | 0.00 | 0.33 | 0.00 |
| 55 | 0.00 | 0.00 | 0.14 | 0.14 | 0.00 | 0.00 | 0.43 | 0.14 |
| 56 | 0.00 | 0.14 | 0.14 | 0.14 | 0.29 | 0.29 | 0.57 | 0.29 |
| 57 | 0.00 | 0.00 | 0.14 | 0.14 | 0.14 | 0.14 | 0.57 | 0.43 |
| 58 | 0.00 | 0.29 | 0.14 | 0.14 | 0.43 | 0.43 | 0.57 | 0.57 |
| 59 | 0.00 | 0.00 | 0.00 | 0.29 | 0.29 | 0.14 | 0.43 | 0.57 |
| 60 | 0.14 | 0.29 | 0.00 | 0.14 | 0.43 | 0.29 | 0.43 | 0.43 |
| 61 | 0.14 | 0.00 | 0.00 | 0.29 | 0.29 | 0.14 | 0.29 | 0.43 |
| 62 | 0.43 | 0.00 | 0.00 | 0.14 | 0.43 | 0.14 | 0.14 | 0.00 |

Table A (continued).

| Trap ID | Occasion | | | | | | | |
| --- | --- | --- | --- | --- | --- | --- | --- | --- |
|  | 1 | 2 | 3 | 4 | 5 | 6 | 7 | 8 |
| 63 | 0.14 | 0.00 | 0.00 | 0.14 | 0.29 | 0.29 | 0.14 | 0.29 |
| 64 | 0.33 | 0.00 | 0.00 | 0.33 | 0.50 | 0.33 | 0.17 | 0.33 |
| 65 | 0.25 | 0.00 | 0.00 | 0.00 | 0.25 | 0.50 | 0.00 | 0.25 |
| 66 | 0.25 | 0.25 | 0.25 | 0.25 | 0.50 | 0.25 | 0.25 | 0.25 |
| 67 | 0.17 | 0.33 | 0.17 | 0.50 | 0.50 | 0.33 | 0.50 | 0.33 |
| 68 | 0.29 | 0.43 | 0.14 | 0.43 | 0.29 | 0.14 | 0.29 | 0.29 |
| 69 | 0.29 | 0.43 | 0.29 | 0.14 | 0.43 | 0.29 | 0.29 | 0.14 |
| 70 | 0.29 | 0.43 | 0.14 | 0.29 | 0.14 | 0.14 | 0.29 | 0.29 |
| 71 | 0.14 | 0.43 | 0.43 | 0.14 | 0.14 | 0.14 | 0.14 | 0.29 |
| 72 | 0.14 | 0.43 | 0.29 | 0.00 | 0.43 | 0.43 | 0.29 | 0.29 |
| 73 | 0.00 | 0.29 | 0.43 | 0.00 | 0.43 | 0.43 | 0.14 | 0.29 |
| 74 | 0.00 | 0.29 | 0.29 | 0.00 | 0.43 | 0.43 | 0.43 | 0.29 |
| 75 | 0.00 | 0.14 | 0.29 | 0.00 | 0.29 | 0.29 | 0.00 | 0.14 |
| 76 | 0.00 | 0.14 | 0.29 | 0.00 | 0.29 | 0.14 | 0.14 | 0.00 |
| 77 | 0.00 | 0.00 | 0.17 | 0.00 | 0.17 | 0.00 | 0.17 | 0.00 |
| 78 | 0.00 | 0.00 | 0.25 | 0.00 | 0.25 | 0.00 | 0.00 | 0.00 |
| 79 | 0.00 | 0.00 | 0.25 | 0.00 | 0.00 | 0.00 | 0.25 | 0.00 |
| 80 | 0.00 | 0.17 | 0.00 | 0.00 | 0.17 | 0.00 | 0.33 | 0.17 |
| 81 | 0.00 | 0.00 | 0.14 | 0.00 | 0.14 | 0.14 | 0.14 | 0.14 |
| 82 | 0.00 | 0.14 | 0.00 | 0.14 | 0.29 | 0.29 | 0.14 | 0.43 |
| 83 | 0.00 | 0.00 | 0.29 | 0.14 | 0.14 | 0.14 | 0.00 | 0.14 |
| 84 | 0.14 | 0.00 | 0.14 | 0.29 | 0.29 | 0.29 | 0.00 | 0.43 |
| 85 | 0.00 | 0.14 | 0.43 | 0.29 | 0.43 | 0.14 | 0.00 | 0.14 |
| 86 | 0.29 | 0.43 | 0.14 | 0.43 | 0.29 | 0.00 | 0.00 | 0.14 |
| 87 | 0.14 | 0.29 | 0.29 | 0.29 | 0.43 | 0.14 | 0.14 | 0.14 |
| 88 | 0.14 | 0.29 | 0.00 | 0.29 | 0.43 | 0.43 | 0.29 | 0.14 |
| 89 | 0.14 | 0.29 | 0.14 | 0.29 | 0.29 | 0.43 | 0.43 | 0.14 |
| 90 | 0.33 | 0.17 | 0.00 | 0.33 | 0.33 | 0.67 | 0.50 | 0.17 |
| 91 | 0.00 | 0.25 | 0.25 | 0.25 | 0.25 | 0.75 | 0.75 | 0.25 |
| 92 | 0.25 | 0.25 | 0.25 | 0.25 | 0.25 | 1.00 | 0.50 | 0.25 |
| 93 | 0.33 | 0.17 | 0.17 | 0.33 | 0.50 | 0.83 | 0.33 | 0.67 |
| 94 | 0.43 | 0.29 | 0.14 | 0.29 | 0.14 | 0.57 | 0.29 | 0.43 |
| 95 | 0.29 | 0.29 | 0.00 | 0.14 | 0.29 | 0.57 | 0.43 | 0.57 |
| 96 | 0.43 | 0.43 | 0.00 | 0.29 | 0.14 | 0.29 | 0.29 | 0.57 |
| 97 | 0.57 | 0.57 | 0.00 | 0.29 | 0.43 | 0.29 | 0.29 | 0.57 |
| 98 | 0.14 | 0.43 | 0.00 | 0.43 | 0.29 | 0.29 | 0.14 | 0.43 |
| 99 | 0.57 | 0.29 | 0.14 | 0.14 | 0.29 | 0.57 | 0.43 | 0.57 |
| 100 | 0.29 | 0.29 | 0.00 | 0.14 | 0.29 | 0.29 | 0.14 | 0.43 |
| 101 | 0.29 | 0.14 | 0.29 | 0.00 | 0.29 | 0.29 | 0.29 | 0.29 |
| 102 | 0.14 | 0.14 | 0.14 | 0.00 | 0.14 | 0.29 | 0.14 | 0.29 |
| 103 | 0.17 | 0.17 | 0.17 | 0.00 | 0.33 | 0.33 | 0.33 | 0.33 |
| 104 | 0.00 | 0.25 | 0.25 | 0.00 | 0.25 | 0.00 | 0.25 | 0.25 |
| 105 | 0.00 | 0.00 | 0.25 | 0.00 | 0.25 | 0.25 | 0.50 | 0.50 |
| 106 | 0.17 | 0.00 | 0.17 | 0.33 | 0.67 | 0.50 | 0.50 | 0.33 |
| 107 | 0.43 | 0.00 | 0.29 | 0.14 | 0.29 | 0.43 | 0.57 | 0.29 |
| 108 | 0.29 | 0.00 | 0.14 | 0.43 | 0.29 | 0.29 | 0.43 | 0.14 |
| 109 | 0.57 | 0.14 | 0.14 | 0.43 | 0.43 | 0.43 | 0.43 | 0.14 |
| 110 | 0.57 | 0.14 | 0.29 | 0.43 | 0.43 | 0.57 | 0.57 | 0.14 |
| 111 | 0.43 | 0.29 | 0.29 | 0.29 | 0.57 | 0.43 | 0.57 | 0.29 |
| 112 | 0.43 | 0.29 | 0.14 | 0.29 | 0.57 | 0.29 | 0.29 | 0.29 |
| 113 | 0.43 | 0.29 | 0.14 | 0.14 | 0.57 | 0.57 | 0.43 | 0.57 |
| 114 | 0.14 | 0.14 | 0.14 | 0.00 | 0.71 | 0.57 | 0.14 | 0.43 |
| 115 | 0.29 | 0.14 | 0.14 | 0.14 | 0.43 | 0.57 | 0.14 | 0.43 |
| 116 | 0.00 | 0.00 | 0.17 | 0.17 | 0.67 | 0.83 | 0.17 | 0.33 |
| 117 | 0.00 | 0.00 | 0.25 | 0.25 | 0.50 | 1.00 | 0.25 | 0.25 |
| 118 | 0.00 | 0.00 | 0.33 | 0.00 | 0.67 | 1.00 | 0.33 | 0.67 |
| 119 | 0.00 | 0.00 | 0.20 | 0.00 | 0.60 | 0.60 | 0.20 | 0.20 |
| 120 | 0.20 | 0.00 | 0.20 | 0.00 | 0.60 | 0.40 | 0.20 | 0.20 |
| 121 | 0.20 | 0.00 | 0.20 | 0.20 | 0.40 | 0.40 | 0.20 | 0.20 |
| 122 | 0.40 | 0.00 | 0.20 | 0.60 | 0.40 | 0.40 | 0.20 | 0.00 |
| 123 | 0.20 | 0.00 | 0.20 | 0.40 | 0.60 | 0.60 | 0.40 | 0.20 |
| 124 | 0.00 | 0.00 | 0.33 | 0.33 | 0.67 | 0.33 | 0.33 | 0.33 |

**Table B. Results of the model selection done for May and June using secr** (Efford, 2016). The best models are shown in bold, and included differences in σ between sexes, temporal trend (May) and temporal trend and differences between sexes (June) for λ_0_.

| MAY | | | | | | | | |
| --- | --- | --- | --- | --- | --- | --- | --- | --- |
| Model | | |  |  |  |  |  |  |
| D | g0 | sigma | npar | logLik | AIC | AICc | dAICc | AICcwt |
| **~1** | **~T** | **~h2** | **6** | **-414.29** | **840.59** | **841.72** | **0.00** | **0.76** |
| ~1 | ~T+h2 | ~h2 | 7 | -414.24 | 842.48 | 844.01 | 2.29 | 0.24 |
| ~1 | ~b | ~h2 | 6 | -424.87 | 861.74 | 862.88 | 21.16 | 0.00 |
| ~1 | ~1 | ~h2 | 5 | -440.31 | 890.63 | 891.43 | 49.70 | 0.00 |
| ~1 | ~h2 | ~h2 | 6 | -440.25 | 892.50 | 893.64 | 51.91 | 0.00 |
| ~1 | ~1 | ~1 | 4 | -445.61 | 899.22 | 899.74 | 58.02 | 0.00 |
| JUNE | | | | | | | | |
| Model | | |  |  |  |  |  |  |
| D | g0 | sigma | npar | logLik | AIC | AICc | dAICc | AICcwt |
| **~1** | **~T+h2** | **~h2** | **7** | **-910.20** | **1834.41** | **1835.22** | **0.00** | **0.88** |
| ~1 | ~T | ~h2 | 6 | -913.47 | 1838.95 | 1839.55 | 4.33 | 0.10 |
| ~1 | ~h2 | ~h2 | 6 | -915.28 | 1842.57 | 1843.17 | 7.95 | 0.02 |
| ~1 | ~1 | ~h2 | 5 | -918.47 | 1846.94 | 1847.37 | 12.15 | 0.00 |
| ~1 | ~1 | ~1 | 4 | -934.89 | 1877.78 | 1878.07 | 42.84 | 0.00 |
