# Supplementary material for: Spatial capture-recapture design and modelling for the study of small mammals
Source: PLoS One. 2018 Jun 7;13(6):e0198766. doi: 10.1371/journal.pone.0198766 (PMC5991742; doi:10.1371/journal.pone.0198766)
Supplement: S2 Supporting Information — (HTML) [file pone.0198766.s002.html]

Spatial capture-recapture design and modelling for the study of small mammals


# Spatial capture-recapture design and modelling for the study of small mammals

### *R + Nimble code for May*

#### *December, 3 2107*

#### **Juan Romairone\(^1\)**, **José Jiménez\(^2\)**, **Juan José Luque-Larena\(^1\)\(^,\)\(^3\)**, **François Mougeot\(^2\)**

\(^1\) Ciencias Agroforestales, Escuela Técnica Superior de Ingenierías, Universidad de Valladolid, Avda. De Madrid 44, 34004, Palencia, Spain  
\(^2\) Instituto de Investigación en Recursos Cinegéticos (IREC, CSIC-UCLM-JCCM), Ronda de Toledo 12, 13071 Ciudad Real, Spain.  
\(^3\) Instituto Universitario de Investigación en Gestión Forestal Sostenible.

### Define working directory

```
setwd('C:/Users/Josele/OneDrive/Topillos/01 MayoV1/DEF/DEF//TopillosMayo')
```

### Data

Load data, constants and inits to use in nimble:

```
load("dataMay.Rdata")
load("constantsMay.RData")
load("initsMay.RData")
```

### Code

BUGS code in Nimble

```
library(nimble)
```

```
## nimble version 0.6-7 is loaded.
## For more information on NIMBLE and a User Manual,
## please visit http://R-nimble.org.
```

```
## 
## Attaching package: 'nimble'
```

```
## The following object is masked from 'package:stats':
## 
##     simulate
```

```
## define the model
code <- nimbleCode({
  psi ~ dunif(0,1)     # prior for data augmentation parameter
  pi  ~ dunif(0,1)     # prior por sex
  alpha0 ~ dnorm(0,.01) # prior for intercept in time covariate
  alpha2 ~ dnorm(0,.01) # prior for slope in time covariate

  for(t in 1:2){
    sigma[t]~dunif(0, 20) # prior for sigma by sex
    alpha1[t]<-1/(2*(sigma[t]*sigma[t])) # sigma parametrisation
  }

  for(i in 1:M){
    z[i] ~ dbern(psi)
    SEX[i]~dbern(pi)
    SEX2[i]<-SEX[i] + 1
    s[i,1] ~ dunif(xlim[1],xlim[2])
    s[i,2] ~ dunif(ylim[1],ylim[2])
    # Euclidean distance between activity centers and traps
    d2[i,1:ntraps] <- pow(pow(s[i,1]-X[1:ntraps,1],2) + pow(s[i,2]-X[1:ntraps,2],2),1) 

    for(k in 1:K){
      logit(p0[k])<- alpha0 + alpha2*time[k] # p0 covariate by time
      lp[i,k,1:ntraps] <- p0[k]*exp(-alpha1[SEX2[i]]*d2[i,1:ntraps])*z[i]*dead[i,k]
      cp[i,k,1:ntraps] <- lp[i,k,1:ntraps]/(1+sum(lp[i,k,1:ntraps]))
      cp[i,k,ntraps2] <- 1-sum(cp[i,k,1:ntraps])
      Ycat[i,k] ~ dcat(cp[i,k,1:ntraps2])
    }
  }
  N <- sum(z[1:M])                             # Total number
  Nmales <- sum(z[1:M]*SEX[1:M])               # Males
  Nfemales <- sum(z[1:M]*(1-SEX[1:M]))         # Females
  A <- ((xlim[2]-xlim[1]))*((ylim[2]-ylim[1])) # State space size
  D <- 1e4*N/A               # Density (individuals/ha)
  Dmales<-1e4*Nmales/A       # Density (males/ha)
  Dfemales<-1e4*Nfemales/A   # Density (females/ha)
})
```

```
inits<-inits
constants<-constants
data <- data
```

Run the model

```
nimbleOptions(allowDynamicIndexing = TRUE)
Rmodel <- nimbleModel(code=code, constants=constants, data=data, inits=inits, check=FALSE)
```

```
## defining model...
```

```
## building model...
```

```
## setting data and initial values...
```

```
## running calculate on model (any error reports that follow may simply reflect missing values in model variables) ... 
## checking model sizes and dimensions... This model is not fully initialized. This is not an error. To see which variables are not initialized, use model$initializeInfo(). For more information on model initialization, see help(modelInitialization).
## model building finished.
```

```
Cmodel <- compileNimble(Rmodel)
```

```
## compiling... this may take a minute. Use 'showCompilerOutput = TRUE' to see C++ compiler details.
## compilation finished.
```

```
params<-c('alpha0','alpha2','N','Nmales','Nfemales','psi','pi','D','Dmales','Dfemales','sigma')
mcmcSCR<-configureMCMC(Rmodel, monitors=params)

SCRMCMC <- buildMCMC(mcmcSCR)

CompSCRMCMC <- compileNimble(SCRMCMC, project = Rmodel)
```

```
## compiling... this may take a minute. Use 'showCompilerOutput = TRUE' to see C++ compiler details.
## compilation finished.
```

```
samplesList <- runMCMC(CompSCRMCMC, niter =150000, nburnin = 5000, nchains = 3, samplesAsCodaMCMC = TRUE)
```

```
## running chain 1...
```

```
## |-------------|-------------|-------------|-------------|
## |-------------------------------------------------------|
```

```
## running chain 2...
```

```
## |-------------|-------------|-------------|-------------|
## |-------------------------------------------------------|
```

```
## running chain 3...
```

```
## |-------------|-------------|-------------|-------------|
## |-------------------------------------------------------|
```

### Results

```
library(coda)
library(lattice)

summary(mcmc.list(samplesList))
```

```
## 
## Iterations = 1:145000
## Thinning interval = 1 
## Number of chains = 3 
## Sample size per chain = 145000 
## 
## 1. Empirical mean and standard deviation for each variable,
##    plus standard error of the mean:
## 
##              Mean       SD  Naive SE Time-series SE
## D        142.9258 38.50327 0.0583785       1.125481
## Dfemales  96.2285 36.40973 0.0552043       1.148787
## Dmales    46.6972  8.65707 0.0131258       0.102591
## N        367.8909 99.10743 0.1502663       2.896988
## Nfemales 247.6923 93.71863 0.1420958       2.956977
## Nmales   120.1987 22.28330 0.0337859       0.264069
## alpha0    -3.5338  0.31078 0.0004712       0.004251
## alpha2     0.9075  0.13881 0.0002105       0.000579
## pi         0.3431  0.08582 0.0001301       0.002141
## psi        0.3683  0.10000 0.0001516       0.002827
## sigma[1]   5.9446  1.19045 0.0018050       0.022645
## sigma[2]  11.4988  1.49205 0.0022622       0.019859
## 
## 2. Quantiles for each variable:
## 
##              2.5%      25%      50%      75%    97.5%
## D         88.5781 115.3846 135.9751 162.7817 235.0427
## Dfemales  46.6200  70.3186  88.9666 114.6076 184.1492
## Dmales    33.0225  40.4040  45.4545  51.6706  66.4336
## N        228.0000 297.0000 350.0000 419.0000 605.0000
## Nfemales 120.0000 181.0000 229.0000 295.0000 474.0000
## Nmales    85.0000 104.0000 117.0000 133.0000 171.0000
## alpha0    -4.1568  -3.7395  -3.5289  -3.3217  -2.9399
## alpha2     0.6432   0.8127   0.9042   0.9990   1.1875
## pi         0.1888   0.2808   0.3393   0.4007   0.5183
## psi        0.2255   0.2973   0.3504   0.4207   0.6058
## sigma[1]   4.0760   5.0910   5.7864   6.6339   8.6624
## sigma[2]   9.0304  10.4405  11.3428  12.3856  14.8672
```

```
xyplot(mcmc.list(samplesList))
```

```
samplesn<-rbind(as.matrix(samplesList[1]),as.matrix(samplesList[2]),as.matrix(samplesList[3]))
par(mfrow=c(1,2))
hist(window(samplesn[,'sigma[1]'],start=5000), main='Females', xlab=expression(sigma))
hist(window(samplesn[,'sigma[2]'],start=5000), main='Males', xlab=expression(sigma))
```
