# Supplementary material for: Spatial capture-recapture design and modelling for the study of small mammals
Source: PLoS One. 2018 Jun 7;13(6):e0198766. doi: 10.1371/journal.pone.0198766 (PMC5991742; doi:10.1371/journal.pone.0198766)
Supplement: S3 Supporting Information — (HTML) [file pone.0198766.s003.html]

Spatial capture-recapture design and modelling for the study of small mammals


# Spatial capture-recapture design and modelling for the study of small mammals

### *R + Nimble code for June*

#### *December, 3 2107*

#### **Juan Romairone\(^1\)**, **José Jiménez\(^2\)**, **Juan José Luque-Larena\(^1\)\(^,\)\(^3\)**, **François Mougeot\(^2\)**

\(^1\) Ciencias Agroforestales, Escuela Técnica Superior de Ingenierías, Universidad de Valladolid, Avda. De Madrid 44, 34004, Palencia, Spain  
\(^2\) Instituto de Investigación en Recursos Cinegéticos (IREC, CSIC-UCLM-JCCM), Ronda de Toledo 12, 13071 Ciudad Real, Spain.  
\(^3\) Instituto Universitario de Investigación en Gestión Forestal Sostenible.

### Define working directory

```
setwd('C:/Users/Jose/OneDrive/Topillos/02 Junio/DEF/DEF')
```

### Data

Load data, constants and inits to use in nimble:

```
load("dataJun.Rdata")
load("constantsJun.RData")
load("initsJun.RData")
```

### Code

BUGS code in Nimble

```
library(nimble)
```

```
## nimble version 0.6-9 is loaded.
## For more information on NIMBLE and a User Manual,
## please visit http://R-nimble.org.
```

```
## 
## Attaching package: 'nimble'
```

```
## The following object is masked from 'package:stats':
## 
##     simulate
```

```
## define the model
code <- nimbleCode({
  psi ~ dunif(0,1)
  pi  ~ dunif(0,1)
  alpha2 ~ dnorm(0,.1)

  for(t in 1:2){
    alpha0[t] ~ dnorm(0,.1)   
    for(k in 1:K){  
      logit(p0[t,k])<- alpha0[t] + alpha2*time[k]
    }  
    sigma[t]~dunif(0, 20)
    alpha1[t]<-1/(2*(sigma[t]*sigma[t]))
  }

  for(i in 1:M){
    z[i] ~ dbern(psi)
    SEX[i]~dbern(pi)
    SEX2[i]<-SEX[i] + 1
    s[i,1] ~ dunif(xlim[1],xlim[2])
    s[i,2] ~ dunif(ylim[1],ylim[2])
    d2[i,1:ntraps] <- pow(pow(s[i,1]-X[1:ntraps,1],2) + pow(s[i,2]-X[1:ntraps,2],2),1)

    for(k in 1:K){
      lp[i,k,1:ntraps] <- p0[SEX2[i],k]*exp(-alpha1[SEX2[i]]*d2[i,1:ntraps])*z[i]*dead[i,k]
      cp[i,k,1:ntraps] <- lp[i,k,1:ntraps]/(1+sum(lp[i,k,1:ntraps]))
      cp[i,k,ntraps2] <- 1-sum(cp[i,k,1:ntraps])  # Última celda = no capturado
      Ycat[i,k] ~ dcat(cp[i,k,1:ntraps2])
    }
  }
  N <- sum(z[1:M])
  Nmales <- sum(z[1:M]*SEX[1:M])
  Nfemales <- sum(z[1:M]*(1-SEX[1:M]))
  A <- ((xlim[2]-xlim[1]))*((ylim[2]-ylim[1]))
  D <- 1e4*N/A
  Dmales<-1e4*Nmales/A
  Dfemales<-1e4*Nfemales/A
})
```

Data and inits

```
inits<-inits
constants<-constants
data <- data
```

Run the model

```
Rmodel <- nimbleModel(code=code, constants=constants, data=data, inits=inits, check=FALSE)
```

```
## defining model...
```

```
## building model...
```

```
## setting data and initial values...
```

```
## running calculate on model (any error reports that follow may simply reflect missing values in model variables) ... 
## checking model sizes and dimensions... This model is not fully initialized. This is not an error. To see which variables are not initialized, use model$initializeInfo(). For more information on model initialization, see help(modelInitialization).
## model building finished.
```

```
Cmodel <- compileNimble(Rmodel)
```

```
## compiling... this may take a minute. Use 'showCompilerOutput = TRUE' to see C++ compiler details.
## compilation finished.
```

```
mcmcSCR<-configureMCMC(Rmodel, monitors=c('alpha0','alpha2','N','Nmales','Nfemales','psi','pi','D','Dmales','Dfemales','sigma'))

SCRMCMC <- buildMCMC(mcmcSCR)

CompSCRMCMC <- compileNimble(SCRMCMC, project = Rmodel)
```

```
## compiling... this may take a minute. Use 'showCompilerOutput = TRUE' to see C++ compiler details.
## compilation finished.
```

```
samplesList <- runMCMC(CompSCRMCMC, niter = 150000, nburnin = 5000, nchains = 3, samplesAsCodaMCMC = TRUE)
```

```
## running chain 1...
```

```
## |-------------|-------------|-------------|-------------|
## |-------------------------------------------------------|
```

```
## running chain 2...
```

```
## |-------------|-------------|-------------|-------------|
## |-------------------------------------------------------|
```

```
## running chain 3...
```

```
## |-------------|-------------|-------------|-------------|
## |-------------------------------------------------------|
```

### Results

```
library(coda)
library(lattice)

summary(mcmc.list(samplesList))
```

```
## 
## Iterations = 1:145000
## Thinning interval = 1 
## Number of chains = 3 
## Sample size per chain = 145000 
## 
## 1. Empirical mean and standard deviation for each variable,
##    plus standard error of the mean:
## 
##               Mean       SD  Naive SE Time-series SE
## D         168.2512 15.79462 2.395e-02      0.1648073
## Dfemales  126.3396 14.68347 2.226e-02      0.1615779
## Dmales     41.9116  5.74324 8.708e-03      0.0461913
## N         433.0786 40.65534 6.164e-02      0.4242141
## Nfemales  325.1981 37.79526 5.731e-02      0.4159015
## Nmales    107.8805 14.78309 2.241e-02      0.1188965
## alpha0[1]  -1.9291  0.22770 3.452e-04      0.0022985
## alpha0[2]  -2.7465  0.25952 3.935e-04      0.0022511
## alpha2      0.2800  0.08823 1.338e-04      0.0002917
## pi          0.2514  0.03908 5.926e-05      0.0004052
## psi         0.4332  0.04349 6.593e-05      0.0004601
## sigma[1]    4.0996  0.29054 4.405e-04      0.0028793
## sigma[2]    8.1166  0.79532 1.206e-03      0.0079367
## 
## 2. Quantiles for each variable:
## 
##               2.5%      25%      50%      75%    97.5%
## D         140.6371 156.9542 167.0552 178.3217 202.0202
## Dfemales  101.0101 116.1616 125.0971 135.5866 158.1197
## Dmales     32.2455  37.6845  41.5695  45.4545  54.3901
## N         362.0000 404.0000 430.0000 459.0000 520.0000
## Nfemales  260.0000 299.0000 322.0000 349.0000 407.0000
## Nmales     83.0000  97.0000 107.0000 117.0000 140.0000
## alpha0[1]  -2.3762  -2.0814  -1.9288  -1.7759  -1.4824
## alpha0[2]  -3.2626  -2.9194  -2.7432  -2.5703  -2.2474
## alpha2      0.1084   0.2205   0.2792   0.3391   0.4542
## pi          0.1803   0.2241   0.2497   0.2768   0.3322
## psi         0.3551   0.4027   0.4307   0.4609   0.5249
## sigma[1]    3.5832   3.8966   4.0815   4.2818   4.7232
## sigma[2]    6.7670   7.5563   8.0441   8.5997   9.8643
```

```
xyplot(mcmc.list(samplesList))
```

```
samplesn<-rbind(as.matrix(samplesList[1]),as.matrix(samplesList[2]),as.matrix(samplesList[3]))
par(mfrow=c(1,2))
hist(window(samplesn[,'sigma[1]'],start=1000), main='Females', xlab=expression(sigma))
hist(window(samplesn[,'sigma[2]'],start=1000), main='Males', xlab=expression(sigma))
```
