# Supplementary material for: Spatial capture-recapture design and modelling for the study of small mammals
Source: PLoS One. 2018 Jun 7;13(6):e0198766. doi: 10.1371/journal.pone.0198766 (PMC5991742; doi:10.1371/journal.pone.0198766)
Supplement: S4 Supporting Information — (DOCX) [file pone.0198766.s004.docx]

**S4 Supporting Information**

**Simulation study to investigate the effect of trap configuration on modelled parameter precision**

We used a simulation study to investigate the influence of the trapping grid configuration on the parameter estimates abundance and sigma. We randomly generated the locations of individual voles using the sigma value estimated with the June model outputs, and a simulated population size of 235 individuals, ie. slightly above the density estimated in June. For simulations, we used either the trap configuration used in May or the one used in June (see Fig 1A and 1B). We used a script modified from Royle *et al.* (2014) and did four separate simulations (for each sex and trap configuration) using the Nimble software, with 100 simulated populations in each case, 5000 iterations and a 1000 burn-in (see the R code below). We used a basal detection rate alpha0=-2, see R code below) and a movement parameter that varied according to sex, as in the June model output (σ_males_=8.1; σ_females_=4.1 m). We determined the error in the estimates obtained for population size (*N̂*) and sigma ($\sigma$) calculating the root mean square error (RMSE) of these parameters for each sex and trap configuration.

The parameters for each simulation were as follows:

Scenario 1: only males -trap configuration of May (12m); N=235; alpha0=-2; $\sigma$_m_=8.1)

Scenario 2: only females -trap configuration of May (12m); N=235; alpha0=-2; $\sigma$_f_=4.1)

Scenario 3: only males -trap configuration of June (9.6m); N=235; alpha0=-2; $\sigma$_m_=8.1)

Scenario 4: only females -trap configuration of June (9.6m); N=235; alpha0=-2; $\sigma$_f_=4.1)

The results (Table C; Figure G) show a greater precision in the parameters estimated using the trap configuration used in June than with the one used in May. This was particularly marked for the simulated female populations (greater RMSE ($\hat{N}$)) because of their lower sigma (Figure H) and lower number of capture events (Table C). With the trap configuration of May, detecting females in more than one trap was more difficult because their average movement distance was equal or less than the minimum distance between traps (Figure H). The RMSE of the sigma parameter was 22% larger with the May trap configuration. The average number of capture events in females was 39.7% greater with the June trap configuration than with the May trap configuration. Overall, the population estimate *N̂* was less precise (greater RMSE [28]).

Table C. Posterior results and RMSE values from 4 sets of 100 simulations under different scenarios (traps configuration of May vs June, for males -M- vs females-F-) using a population size and sigma parameters as estimated for June.

| Trap configuration | Minimum distance between traps (meters) | sex | Mean N. of capture events | N̂ | RMSE(N̂) | σ̂ | RMSE (σ̂) |
| --- | --- | --- | --- | --- | --- | --- | --- |
| May | 12.00 | M | 295.77 | 238.21 | 11.56 | 8.08 | 0.31 |
|  | 12.00 | F | 102.25 | 269.12 | 71.74 | 4.08 | 0.42 |
| June | 9.60 | M | 442.62 | 235.83 | 10.10 | 8.08 | 0.26 |
|  | 9.60 | F | 169.67 | 242.68 | 29.35 | 4.05 | 0.33 |


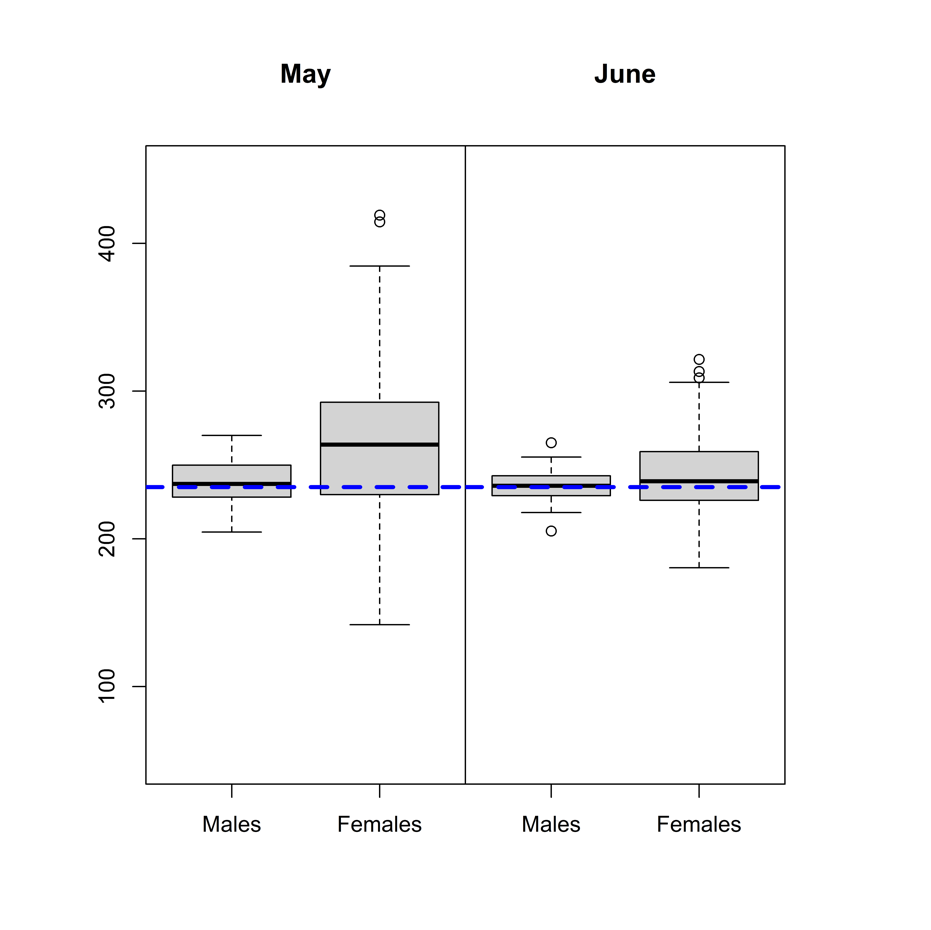


Figure G. Population size estimates from the simulated scenarios (traps configuration used in May- left- or in June - right).


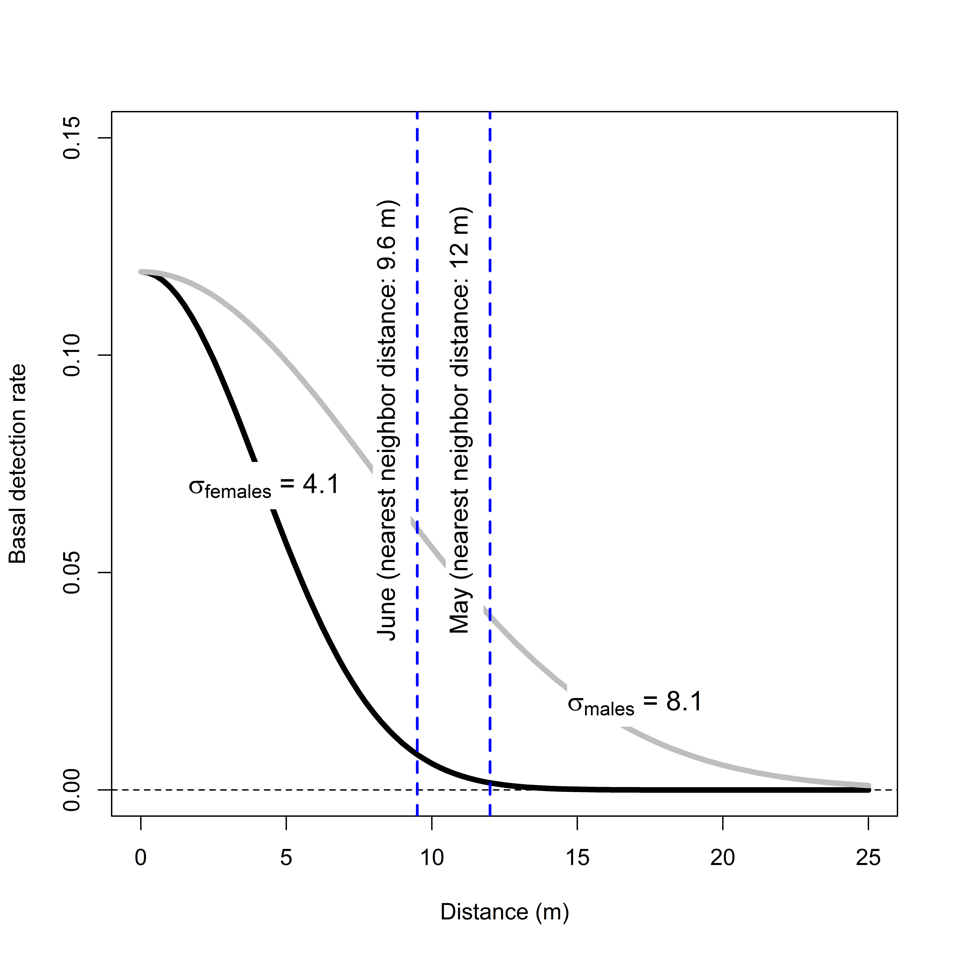


Figure H. Comparison of the sigma parameters of males and females with the minimum distance between traps in the configurations used in May and June. Note that the basal detection rate is very low at the 12m distance for females (black line).

R+BUGS Code. Population simulator and parameters estimate under a multinomial model using Nimble.

library(scrbook)

*# May*

X<-read.table("trapsMay.txt", header=FALSE)

*# June*

*# X<-read.table("trapsJune.txt", header=FALSE)*

X<-data.matrix(X[,2:3])

simMnSCRv2 <-function (N=N, sigma=4.08, alpha0=-2, K = 8, ssbuff = 15.25, rnd = 2013)

{

set.seed(rnd)

traplocs <- X

Dmat <- e2dist(traplocs, traplocs)

ntraps <- nrow(traplocs)

plot(traplocs, pch="+", asp=1)

delta <- ssbuff

Xl <- min(traplocs[, 1] - delta)

Xu <- max(traplocs[, 1] + delta)

Yl <- min(traplocs[, 2] - delta)

Yu <- max(traplocs[, 2] + delta)

A<-(Xu-Xl)*(Yu-Yl)

N <- N

sx <- runif(N, Xl, Xu)

sy <- runif(N, Yl, Yu)

points(sx,sy, pch=16, col="red")

S <- cbind(sx, sy)

D <- e2dist(S, traplocs)

sigma <- sigma

alpha0 <- alpha0

alpha1 <- 1/(2 * sigma * sigma)

alpha2 <- 0

Ycat <- matrix(NA, nrow = N, ncol = K)

Xlag <- matrix(0, nrow = N, ncol = K + 1)

Xlag[, 1] <- rep(0, N)

for (i in 1:N) {

for (k in 1:K) {

lp <- alpha0 + alpha2 * Xlag[i, k] - alpha1 * D[i,

] * D[i, ]

cp <- exp(c(lp, 0))

cp <- cp/sum(cp)

Ycat[i, k] <- sample(1:(ntraps + 1), 1, prob = cp)

if (Ycat[i, k] <= ntraps)

Xlag[i, (k + 1):ncol(Xlag)] <- 1

}

}

captured <- apply(Ycat <= ntraps, 1, sum)

captured <- captured > 0

Ycat <- Ycat[captured, ]

Xlag <- Xlag[captured, ]

reencounter = Xlag[, 1:K]

X <- traplocs

K <- ncol(reencounter)

S1 <- S[captured, ]

S2 <- S[!captured, ]

S <- rbind(S1, S2)

list(Ycat = Ycat, X = traplocs, reencounter = reencounter,

X = X, ssbuff = ssbuff, S = S1,

xlim = c(Xl, Xu), ylim = c(Yl, Yu), A=A,

K = K)

}

out<- matrix(NA,nrow=100,ncol=6)

for(sim in 1:100){

*# Females*

data<-simMnSCRv2(N=235, sigma=4.08, alpha0=-2, K = 8,

ssbuff = 15.25, rnd=(sim + 100))

*# Males*

*# data<-simMnSCRv2(N=235, sigma=8.11, alpha0=-2, K = 8,*

*# ssbuff = 20.2915, rnd=(sim + 100))*

nind<-nrow(data$Ycat)

*# data augmentation*

M<-1000

Ycat<-rbind(data$Ycat,matrix(nrow(data$X)+1,nrow=(M-nind),ncol=data$K))

Sst<-rbind(data$S,cbind(runif(M-nind,data$xlim[1],data$xlim[2]),

runif(M-nind,data$ylim[1],data$ylim[2])))

*# starting values*

zst<-c(rep(1,nind),rep(0,M-nind))

inits <- list (z=zst, sigma=runif(1,.5,1) ,S=Sst)

library(nimble)

*## define the model*

code <- nimbleCode({

psi ~ dunif(0,1)

alpha0 ~ dnorm(0,.1)

logit(p0)<- alpha0

sigma~ dunif(0, 20)

alpha1<-1/(2*(sigma*sigma))

for(i in 1:M){

z[i] ~ dbern(psi)

S[i,1] ~ dunif(xlim[1],xlim[2])

S[i,2] ~ dunif(ylim[1],ylim[2])

d2[i,1:ntraps] <- pow(pow(S[i,1]-X[1:ntraps,1],2) + pow(S[i,2]-X[1:ntraps,2],2),1)

for(k in 1:K){

lp[i,k,1:ntraps] <- p0*exp(-alpha1*d2[i,1:ntraps])*z[i]

cp[i,k,1:ntraps] <- lp[i,k,1:ntraps]/(1+sum(lp[i,k,1:ntraps]))

cp[i,k,ntraps2] <- 1-sum(cp[i,k,1:ntraps])

Ycat[i,k] ~ dcat(cp[i,k,1:ntraps2])

}

}

N <- sum(z[1:M])

A <- (xlim[2]-xlim[1])*(ylim[2]-ylim[1])

D <- 1e4*N/A

})

ntraps2<-nrow(data$X)+1

constants<-list(K=data$K, M=M, ntraps=nrow(data$X), ntraps2=ntraps2)

data <- list (X=data$X, Ycat=Ycat, ylim=data$ylim, xlim=data$xlim)

*# Parameters to monitor*

params <- c('D', 'N', 'alpha0','sigma')

Rmodel <- nimbleModel(code=code, constants=constants, data=data, inits=inits)

Cmodel <- compileNimble(Rmodel)

mcmscr<-configureMCMC(Rmodel, monitors=params)

scrMCMC <- buildMCMC(mcmscr)

SCRMCMC <- compileNimble(scrMCMC, project = Rmodel)

SCRMCMC$run(5000)

samples <- as.matrix(SCRMCMC$mvSamples)

samples<-samples[1000:5000,]

int<-as.numeric(c(quantile(samples[,2],c(0.025,0.975))[1],

quantile(samples[,2],c(0.025,0.975))[2]))

cover95<-int[1]<=235 & int[2]>=235

data$Ycat[data$Ycat==71]<-0 *# May*

*# data$Ycat[data$Ycat==125]<-0 # June*

data$Ycat[data$Ycat>0]<-1

events<-sum(data$Ycat)

out[sim,]<- c(events, mean(samples[,1]),mean(samples[,2]),

mean(samples[,3]),mean(samples[,4]),cover95)

print(out[sim,])

}
